# The KAT module of the SAGA complex maintains the oncogenic gene expression program in *MYCN-*amplified neuroblastoma

**DOI:** 10.1126/sciadv.adm9449

**Published:** 2024-05-31

**Authors:** Clare F. Malone, Nathaniel W. Mabe, Alexandra B. Forman, Gabriela Alexe, Kathleen L. Engel, Ying-Jiun C. Chen, Melinda Soeung, Silvi Salhotra, Allen Basanthakumar, Bin Liu, Sharon Y. R. Dent, Kimberly Stegmaier

**Affiliations:** ^1^ Department of Pediatric Oncology, Dana-Farber Cancer Institute, Boston, MA, USA.; ^2^ Broad Institute of MIT and Harvard, Cambridge, MA, USA.; ^3^ Harvard Medical School, Boston, MA, USA.; ^4^ Department of Epigenetics and Molecular Carcinogenesis, The University of Texas MD Anderson Cancer Center, Houston, TX, USA.; ^5^ The Center for Cancer Epigenetics, The University of Texas MD Anderson Cancer Center, Houston, TX, USA.; ^6^ Division of Hematology/Oncology, Boston Children’s Hospital, Boston, MA, USA.

## Abstract

Pediatric cancers are frequently driven by genomic alterations that result in aberrant transcription factor activity. Here, we used functional genomic screens to identify multiple genes within the transcriptional coactivator Spt-Ada-Gcn5-acetyltransferase (SAGA) complex as selective dependencies for *MYCN*-amplified neuroblastoma, a disease of dysregulated development driven by an aberrant oncogenic transcriptional program. We characterized the DNA recruitment sites of the SAGA complex in neuroblastoma and the consequences of loss of SAGA complex lysine acetyltransferase (KAT) activity on histone acetylation and gene expression. We demonstrate that loss of SAGA complex KAT activity is associated with reduced MYCN binding on chromatin, suppression of MYC/MYCN gene expression programs, and impaired cell cycle progression. Further, we showed that the SAGA complex is pharmacologically targetable in vitro and in vivo with a KAT2A/KAT2B proteolysis targeting chimeric. Our findings expand our understanding of the histone-modifying complexes that maintain the oncogenic transcriptional state in this disease and suggest therapeutic potential for inhibitors of SAGA KAT activity in *MYCN*-amplified neuroblastoma.

## Teaser

The SAGA acetyltransferase complex is required to promote neuroblastoma growth and is targetable with a KAT2A/KAT2B PROTAC.

## INTRODUCTION

Pediatric cancers, such as neuroblastoma, are thought to primarily arise because of a failure of cells to differentiate normally during development (*1*). Normal development relies on carefully controlled patterns of gene expression, which are coordinately regulated by transcription factors and histone-modifying complexes. The activity of histone-modifying complexes is critical for maintaining the oncogenic state in cancers, particularly pediatric, and targeting disease-relevant epigenetic regulators can convey antitumor activity (*2*). For example, studies in *KMT2A*-rearranged leukemia identified a critical interaction between the oncofusion protein and chromatin-associated complexes and specifically identified Menin and disruptor of telomeric silencing 1-like (DOT1L) as key mediators of the oncogenic transcriptional program (*3*–*6*). Inhibitors that disrupt the interaction between the lysine methyltransferase 2A (KMT2A) fusion and Menin have entered clinical trials, and reports indicate substantial clinical activity including complete responses (*7*, *8*). Moreover, gold standard evidence of on-target mechanism of action is provided by documentation of resistance mutations in *MEN1* at the time of progression (*9*). These findings in *KMT2A*-rearranged leukemia provide proof of concept that targeting histone-modifying complexes can have therapeutic benefit in cancer. However, identifying the key relevant complexes to target is not trivial because the cooperating epigenetic complexes do not necessarily harbor mutations.

We focused our efforts on identifying cooperating epigenetic complexes in *MYCN*-amplified neuroblastoma. Neuroblastoma, the most common solid tumor of childhood, arises in the developing autonomic nervous system. Despite aggressive treatment of this disease with surgery, radiation, chemotherapy, immunotherapy, stem cell transplants, and differentiation therapy, the 5-year survival for high-risk neuroblastoma is ~50% (*10*). Hence, neuroblastoma accounts for a disproportionately high number of cancer-related deaths in children (*11*). Amplification of the transcription factor *MYCN* occurs in about 20 to 25% of neuroblastoma and is associated with high-risk disease. MYCN itself remains difficult to target directly, so alternative approaches to disrupting the MYCN-oncogenic transcriptional program are needed.

Several epigenetic factors induce differentiation or otherwise impede neuroblastoma cell growth. The histone deacetylases HDAC1 and HDAC2 are critical for maintaining the undifferentiated cell state in neuroblastoma, and inhibition of these HDACs alters the oncogenic transcriptional program and induces differentiation (*12*, *13*). The histone-methylating polycomb repressive complex 2 (PRC2) is an additional critical mediator of gene expression in *MYCN*-amplified neuroblastoma and inhibition of one of the complex members, enhancer of zeste homolog 2 (EZH2), has antitumor activity in some murine models (*14*–*16*). More recently, the histone acetyltransferase EP300 was found to maintain histone acetylation at enhancer sites in neuroblastoma, and small molecule–induced degradation of this target has antitumor activity in neuroblastoma murine models (*17*). These findings demonstrate that histone-modifying complexes are critical to enforcing the oncogenic transcriptional program in neuroblastoma and that targeting the epigenetic factors that maintain the oncogenic cell state could be an effective approach for therapeutic development in this disease as well.

A complete characterization of the specific complexes that control the epigenetic landscape in *MYCN*-amplified neuroblastoma is critical not only for understanding the biology of this disease but also for finding new potential avenues for therapeutic development. Here, we use the Cancer Dependency Map (DepMap) functional genomic screens to identify the lysine acetyltransferase (KAT) module of the Spt-Ada-Gcn5-acetyltransferase (SAGA) complex as an epigenetic regulator important for *MYCN*-amplified neuroblastoma growth.

## RESULTS

### *MYCN*-amplified neuroblastoma is selectively dependent on the SAGA complex

**Fig. 1.**
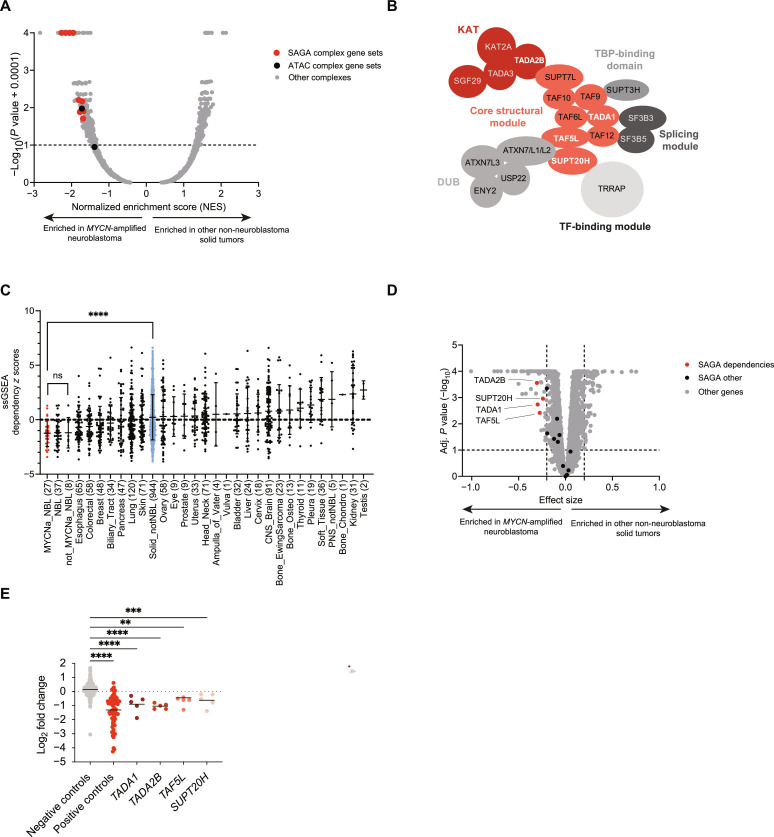
SAGA complex members are selective dependencies in *MYCN*-amplified neuroblastoma. (**A**) Volcano plot depicting normalized enrichment scores (NESs) calculated for epigenetic complexes found in CORUM. Genetic dependencies for *MYCN*-amplified neuroblastoma cell lines were compared to all other non-neuroblastoma solid tumor cell lines. SAGA complex is indicated in red, and ATAC complex is indicated in black. Significance is based on −log_10_(*P*) of >1.0. (**B**) The SAGA complex, adapted from Helmlinger and Tora (*18*). The candidate *MYCN*-amplified neuroblastoma dependency genes in the KAT and core modules are highlighted in white text. (**C**) Means + SD of dot plots depicting the ssGSEA dependency *z* scores for the SAGA complex gene set (*18*) across lineages. *MYCN*-amplified and non–*MYCN*-amplified neuroblastomas are presented as individual lineages. MYCN status for two cells was not determined because of ambiguity in the amplification status and was not included in the subsets. Neuroblastoma includes both the non–*MYCN*- and *MYCN*-amplified subsets. Unpaired *t* test with Welch’s correction, ****P < 0.0001. ns, not significant. (**D**) Volcano plots depicting the 23Q2 CRISPR (DepMap + Score, Chronos) genome-wide differential dependency for *MYCN*-amplified neuroblastoma versus all other non-neuroblastoma solid tumor cell lines. The four SAGA genes included in the (*24*) in vivo validation screen are highlighted red; all other SAGA complex genes are highlighted black. Significance estimated on the basis of the two-class comparison method implemented in DepMap, limma empirical Bayes cutoff; *P* < 0.01. (**E**) Log_2_ fold changes showing sgRNA depletion in tumors versus input for each of the negative controls (including cutting and noncutting sgRNAs; *n* = 1201), positive controls (*n* = 85), and individual SAGA complex members (*n* = 5, each). Each dot represents the average log_2_ fold change for an individual sgRNA as compared to input. An ordinary one-way analysis of variance (ANOVA) was performed, followed by Dunnett’s multiple comparison test. ****P < 0.0001, ***P = 0.0003, and **P = 0.0088

To identify epigenetic modifying complexes that may contribute to the MYCN oncogenic program in neuroblastoma, we calculated CRISPR-Cas9 dependency scores for individual genes in *MYCN*-amplified neuroblastoma cell lines found in the DepMap Project (*n* = 27; table S1) as compared to dependency scores for all other non-neuroblastoma solid tumor cell lines (*n* = 944). Genes were rank-ordered on the basis of differential dependency and analyzed by gene set enrichment analysis (GSEA) using the comprehensive resource of mammalian protein complexes (CORUM) database to prioritize protein complexes that are enriched among *MYCN*-amplified selective dependencies. The SAGA complex scored among the top enriched epigenetic complexes in *MYCN*-amplified neuroblastoma (Fig. 1A and table S2).

The human SAGA complex is made up of several multiprotein modules (Fig. 1B). These modules include two key enzymatic modules: the KAT module and the deubiquitination (DUB) module, as well as a core structural module, a TATA box–binding protein (TBP)–binding module, a transcription factor (TF)–binding module, and a module that contains proteins shared with the human spliceosome factor 3B (SF3B) RNA splicing complex (*18*). The canonical substrates for SAGA include several acetylation sites on histone H3 and other histones, and a ubiquitination site on histone H2B (*19*, *20*). While the SAGA complex is thought to play a key role in controlling gene expression during development, including in neural stem cells, its role in developmental cancers such as neuroblastoma has not been described to date (*21*–*23*).

To confirm that this complex level dependency is enriched in *MYCN*-amplified neuroblastoma, we generated a metagene signature of all SAGA complex members (*18*) and used ssGSEA (single-sample GSEA) to generate a dependency score for the SAGA complex for each solid tumor cell line in the DepMap dataset. Consistent with the differential dependency analysis, we found that *MYCN*-amplified neuroblastoma cell lines are significantly more dependent on SAGA complex genes compared to all other solid tumor lineages (Fig. 1C). While there is only one SAGA complex, the subunits that are included in the definition of SAGA complex vary within the literature. Therefore, we also compared enrichment among all the SAGA complex definitions found in CORUM and found that all of them showed strong enrichment in *MYCN*-amplified neuroblastoma (fig. S1A and table S2).

Among the most enriched differential gene dependencies observed in *MYCN*-amplified neuroblastoma as compared to all other solid tumor lineages were *TADA2B*, *TADA1*, *SUPT20H*, and *TAF5L*, along with several other members of the core and KAT modules (Fig. 1D). We focused on the deubiquitinase and acetyltransferase activity of SAGA because these are the functions that would be the most tractable therapeutically. We found that none of the genes in the DUB module were selective dependencies, while *TADA2B*, a member of the KAT module, was the dependency most selective for *MYCN*-amplified neuroblastoma (Fig. 1D and fig. S1B).

We previously generated a neuroblastoma-specific single-guide RNA (sgRNA) library and performed a series of secondary CRISPR screens using xenografted tumors in mice to prioritize genetic dependencies that translate in vivo (*24*). Four members of the SAGA complex, *TADA2B*, *TAF5L*, *TADA1*, and *SUPT20H* were included in this effort. We analyzed these datasets and observed that sgRNAs targeting all four genes were significantly depleted in tumors relative to control guides (Fig. 1E). Together, these data nominate the SAGA complex as a selective dependency in *MYCN*-amplified neuroblastoma for further investigation.

### TADA2B loss is sufficient to impair SAGA complex histone acetyltransferase activity

Many members of SAGA complex integrate into other complexes, most notably the ADA2A-containing (ATAC) complex. However, *TADA2B*, the strongest outlier dependency in *MYCN*-amplified neuroblastoma, is uniquely found in the SAGA complex and is required for its KAT activity. Its paralog, transcriptional adaptor 2A (TADA2A) or ADA2A (encoded by *TADA2A*), is found in the ATAC complex (*25*). While the ATAC complex showed a moderately enriched dependency in neuroblastoma (Fig. 1A), *TADA2B* displays selectivity for *MYCN*-amplified neuroblastoma in the DepMap dataset, while *TADA2A* is a common essential gene (fig. S1B). Thus, we focused our efforts on TADA2B to specifically interrogate the role of the SAGA complex in *MYCN*-amplified neuroblastoma.

**Fig. 2.**
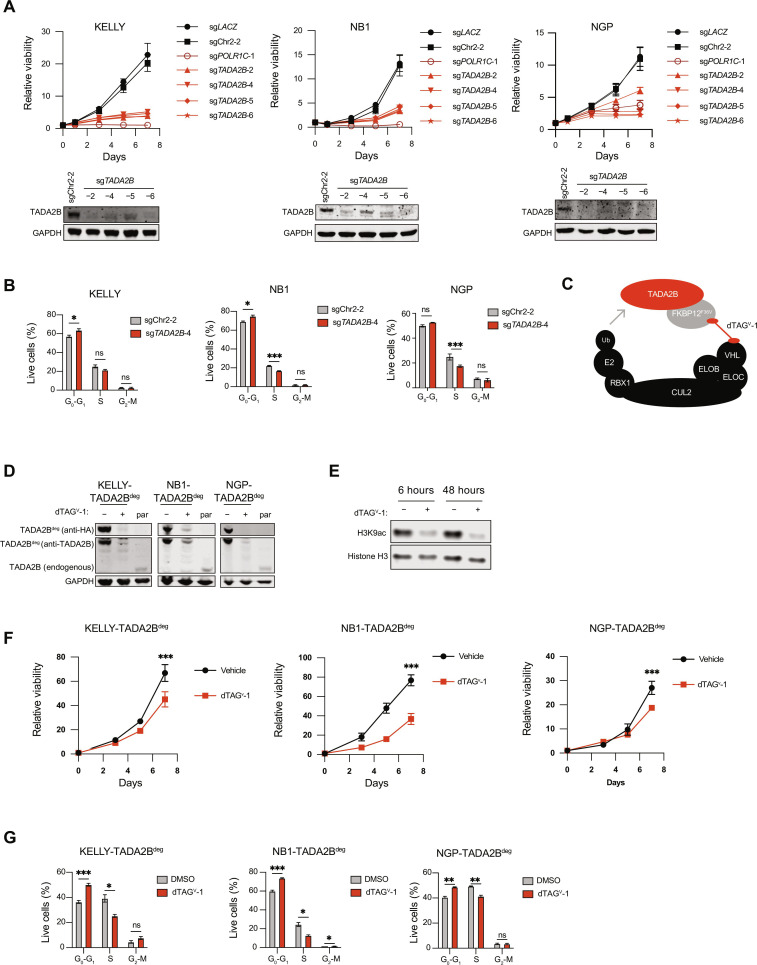
TADA2B is a dependency in *MYCN*-amplified neuroblastoma. (**A**) Cell viability analysis showing growth for neuroblastoma cells infected with sgRNAs targeting negative controls (black), *TADA2B* (red), or the common essential gene *POLR1C* (dark red). Viability was compared to day 0 of plating. Below, Western blots showing TADA2B expression in sgChr2-2 and *TADA2B* KOs. GAPDH is included as a loading control. (**B**) Cell cycle analysis on day 7 for neuroblastoma cells infected with a sgChr2-2 or *TADA2B* sgRNA. Data are shown as mean with SD for three replicates. Significance was determined by multiple unpaired *t* tests with Welch correction for grouped analysis. (**C**) Diagram depicting the utilization of the TADA2B fused to HA-FKBP12^K36V^ model (TADA2B^deg^). (**D**) Western blot showing HA and TADA2B expression in neuroblastoma cells that express TADA2B^deg^ and treated with 500 nM dTAG^V^-1 for 6 hours. GAPDH serves as a loading control. Par stands for parental nonengineered cells. (**E**) KELLY-TADA2B^deg^ cells were treated with DMSO or 500 nM dTAG^V^-1 for 6 or 48 hours as indicated, and then histones were extracted. H3K9ac levels are shown. Histone H3 serves as a loading control. (**F**) Neuroblastoma cells were plated and treated with DMSO or 500 nM dTAG^V^-1 (1 μM dTAG^V^-1 for NGP) for 3, 5, or 7 days, and viability was assessed as compared to day 0. (**G**) Neuroblastoma cells were plated and treated with DMSO or 500 nM dTAG^V^-1 (1 μM dTAG^V^-1 for NGP) for 72 hours, and then an EdU and PI incorporation assay was performed. The bar plot depicts the percentage of cells that were in the indicated stage of the cell cycle. Significance was determined using multiple unpaired *t* tests with Welch correction for grouped analysis. *P < 0.05, **P < 0.01, ***P < 0.001, and ns: *P* > 0.05.

We first confirmed that knockout (KO) of *TADA2B* using multiple independent sgRNAs impaired growth in three *MYCN*-amplified neuroblastoma models in low throughput (Fig. 2A). Impaired growth was not observed with *TADA2B* KO in three pediatric sarcoma solid tumor cell lines, suggesting that *TADA2B* KO is not broadly deleterious (fig. S2A). We also knocked out *TADA2B* in three non–*MYCN*-amplified neuroblastoma cell lines and observed moderately impaired growth in two cell lines, consistent with observations made from DepMap (Fig. 1C and fig. S2B). To determine whether this impaired growth in *MYCN*-amplified neuroblastoma is linked to cell cycle perturbations, we used an 5-ethynyl-2′-deoxyuridine (EdU) and propidium iodide (PI) incorporation assay to assess progression through the cell cycle. We found that neuroblastoma cells with CRISPR-mediated KO of *TADA2B* accumulated in G_0_-G_1_ phase (Fig. 2B).

CRISPR editing is a stochastic event that lacks temporal control, so we next engineered an inducible degradation system for TADA2B to interrogate the direct effects of loss of SAGA KAT activity more precisely. We exogenously expressed *TADA2B* C-terminally tagged with hemagglutinin (HA) and a 12-kDa FK506-binding protein FKBP12 mutant containing an F36V mutation (FKBP12^F36V^) that can be rapidly degraded upon the introduction of dTAG^V^-1, a heterobifunctional molecule that recruits Von Hippel-Lindau (VHL) to the FKBP12^F36V^ tag (Fig. 2C). We then knocked out endogenous *TADA2B* using an sgRNA that does not edit the exogenous *TADA2B* due to a silent mutation we engineered at the protospacer adjacent motif (PAM) site so that the three *MYCN*-amplified cell lines were reliant on the exogenous-tagged TADA2B (fig. S2C). We confirmed that these cell lines had efficient editing of the endogenous *TADA2B* by Tracking of Indels by DEcomposition (TIDE) sequencing and KO of *TADA2B* by Western blotting (Fig. 2D and fig. S2D). The addition of the heterobifunctional molecule dTAG^V^-1 efficiently degraded the exogenous TADA2B (Fig. 2D), and in concordance with TADA2B being required for efficient SAGA KAT activity, we observed a decrease in H3K9 acetylation (H3K9ac) when we degraded TADA2B in all three cell lines (Fig. 2E and fig. S2, E and F). Last, we also observed a reduction in cellular viability with degradation of TADA2B in all three cell lines and an accumulation of cells in G_0_-G_1_ upon degradation of TADA2B, similar to observations seen with CRISPR KO of *TADA2B* (Fig. 2, F and G).

### The SAGA complex is found at promoters of expressed genes

**Fig. 3.**
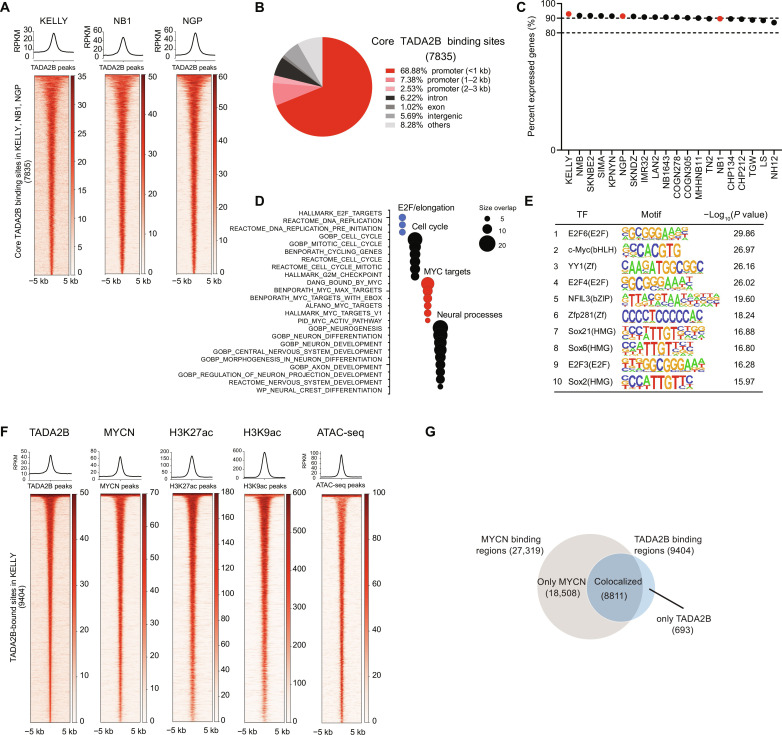
TADA2B colocalizes with MYCN, H3K9Ac, H3K27Ac, and open chromatin at promoters of actively transcribed genes. (**A**) Heatmaps of ChIP-seq TADA2B peak centric signal on the “core” TADA2B binding regions identified on KELLY, NB1, and NGP cell lines. Regions are ranked on the basis of TADA2B signal on KELLY cells. Read density metaplots show average RPKM normalized signal for TADA2B across the core TADA2B peaks in each of the three cell lines. (**B**) Pie chart presenting the genomic distribution of the core TADA2B binding regions. (**C**) Bar plots depicting the percentage of expressed genes among the nearest gene targets for TADA2B core binding regions in a panel of neuroblastoma cell lines. Nearest gene targets for TADA2B core peaks were annotated for hg38 with Homer v4.11. Expressed gene status was estimated as log_2_(TPM + 1) expression of ≥1 based on the Cancer Cell Line Encyclopedia (CCLE) RNA-seq (DepMap 23Q2) data. (**D**) Bubble plot summarizing the functional categories enriched in the nearest gene targets for the TADA2B core peaks. GSEA was performed on the MSigDB v7.4 Collections Hallmarks, c2 and c5 (significance: size overlap ≥ 5 and FDR ≤ 0.05). (**E**) List of top 10 enriched motifs at the promoters of nearest gene targets for the TADA2B core peaks (Homer v4.11, FDR ≤ 1 × 10^−5^). (**F**) Heatmaps depicting the TADA2B, MYCN, H3K27ac, and H3K9ac ChIP-seq and ATAC-seq signal in the TADA2B binding regions identified on KELLY-TADA2B^deg^ cells. Read density metaplots show average RPKM-normalized signal for TADA2B, MYCN, H3K27ac, and H3K9ac across the TADA2B peaks and average RPKM-normalized ATAC-seq signal across the TADA2B peaks. (**G**) Venn diagram showing the overlap of MYCN and TADA2B binding sites as determined by ChIP-seq in KELLY cells.

We next sought to interrogate where in the genome the SAGA complex is recruited in *MYCN*-amplified neuroblastoma, using TADA2B as a proxy for the SAGA complex. Correlation of *TADA2B* dependency with all other gene dependencies in DepMap demonstrated strong codependency with core subunits of SAGA including TATA-box binding protein associated factor 5-like (TAF5L) and SPT20 Homolog, SAGA Complex Component (SUPT20H) (fig. S3A). Further, we confirmed that genetic KO of *TAF5L* significantly reduced cellular viability in *MYCN*-amplified cell lines in a manner similar to *TADA2B* KO (fig. S3B). These data support the use of TADA2B in the KAT domain as a proxy for localization of the SAGA complex, as TADA2B is only found in the SAGA complex. We performed calibrated chromatin immunoprecipitation sequencing (ChIP-seq) with an HA antibody in all three TADA2B^deg^, *MYCN*-amplified neuroblastoma cell lines. To control for off-target HA antibody binding, we performed ChIP-seq 6 hours after treatment with dimethyl sulfoxide (DMSO) or dTAG^V^-1. As expected, treatment with dTAG^V^-1 led to a significant decrease in TADA2B peaks in all three cell lines (fig. S3, C to E). The binding pattern of TADA2B was highly similar across cell lines, and we identified a core binding signature of 7835 genomic peaks that correspond to 6841 neighboring genes, which were shared across three cell lines (Fig. 3A and fig. S3F).

We next analyzed where in the genome these core binding sites were located, and we found that SAGA complex binding is predominantly in the promoter regions (Fig. 3B) consistent with what has previously been described for other SAGA complex members in other cell contexts (*26*). Furthermore, we found that nearly 90% of the genes with SAGA complex binding sites in their promoters were expressed in neuroblastoma cell lines (Fig. 3C). To understand whether there were any functional enrichments among these genes, we performed an overlapping analysis and found that gene sets related to E2F/elongation, cell cycle, MYC targets, neural processes, and regulation of core cellular processes were enriched among genes with TADA2B binding (Fig. 3D). Consistent with this, when we performed motif analysis for promoters with TADA2B binding, we identified E2F, MYC, and neural homeobox motifs among the most enriched sites for TADA2B binding (Fig. 3E and fig. S3G).

Given the observation that *TADA2B* dependency is strongly enriched in *MYCN*-amplified neuroblastoma and that TADA2B occupancy is enriched for the *MYCN* CANNTG motif, we next sought to investigate whether TADA2B colocalizes with MYCN on chromatin. We performed ChIP-seq for MYCN, H3K9ac, and H3K27ac, as well as ATAC sequencing (ATAC-seq) to identify regions of open chromatin. We identified strong colocalization of MYCN, H3K27ac, and H3K9ac at TADA2B binding sites and that areas with TADA2B binding were also generally accessible as determined by ATAC-seq (Fig. 3, F and G). Together, these results support that the SAGA complex, as inferred by TADA2B binding, is predominantly colocalized with MYCN in the promoters of expressed and actively transcribed genes in *MYCN*-amplified neuroblastoma.

### Loss of TADA2B results in global loss of H3K9ac and loss of MYCN and H3K27ac occupancy at TADA2B sites

We observed that TADA2B colocalizes with MYCN, H3K9ac, and H3K27ac at regions of open chromatin. We were next interested in how loss of SAGA complex acetyltransferase activity might alter these binding patterns. To that end, we performed calibrated ChIP-seq for MYCN, H3K9ac, and H3K27ac 6 hours following dTAG^V^-1–mediated degradation of TADA2B and compared the binding patterns of these marks with matched DMSO-treated samples.

**Fig. 4.**
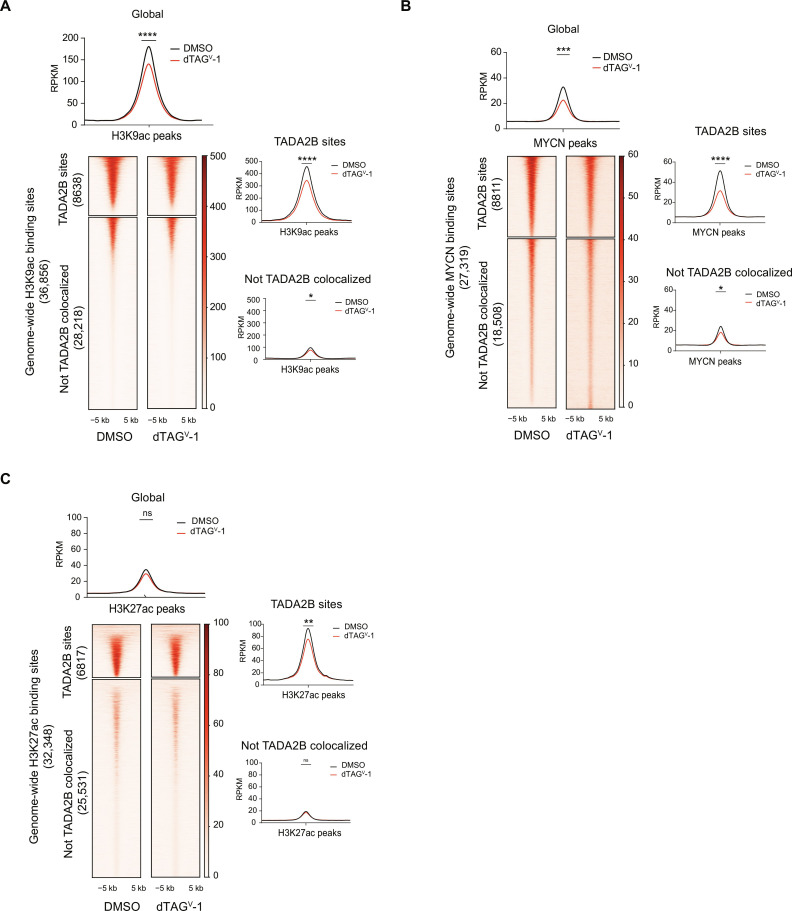
Loss of TADA2B results in rapid loss of H3K9ac and MYCN binding at TADA2B-bound sites. (**A**) Genome-wide heatmaps of H3K9ac in KELLY-TADA2B^deg^ cells treated with DMSO or 500 nM dTAG^V^-1 for 6 hours. Heatmaps are depicted on the universe of H3K9ac-merged binding sites across all conditions and subdivided on the basis of whether peaks are overlapping with TADA2B binding sites. Each region is ranked on the basis of the binding signal in DMSO. At top, read density metaplots showing average RPKM (reads per kilobase per million) normalized signal for H3K9ac across the universe of binding sites depicted below in DMSO-treated (black) and 500 nM dTAG^V^-1–treated (red) KELLY-TADA2B^deg^ cells. To the right, read density metaplots showing average RPKM-normalized signal for H3K9ac for each of TADA2B-bound and nonbound regions depicted by DMSO-treated (black) and 500 nM dTAG^V^-1–treated (red) KELLY-TADA2B^deg^ cells. Differential read density between DMSO and degrader conditions estimated on the basis of unpaired *t* test with Welch’s correction. (**B**) As in (A), for genome-wide heatmaps of MYCN binding in KELLY-TADA2B^deg^ cells treated with DMSO or dTAG^V^-1 for 6 hours. (**C**) As in (A), for genome-wide heatmaps of H3K27ac in KELLY-TADA2B^deg^ cells treated with DMSO or dTAG^V^-1 for 6 hours. ****P < 0.0001, ***P < 0.001, **P < 0.01, and *P < 0.5.

We had previously observed a rapid loss of bulk H3K9ac after TADA2B degradation (Fig. 2E). Consistent with this, we observed decreased H3K9ac 6 hours after treatment with dTAG^V^-1 (Fig. 4A and fig. S4A). This loss of H3K9ac is genome-wide and notably occurs predominately at TADA2B-bound sites (Fig. 4A and fig. S4, A and B). We found that MYCN is decreased at a significant subset of sites 6 hours after dTAG^V^-1 treatment–induced degradation of TADA2B (Fig. 4B and fig. S4C). While this decrement is observed at sites across the genome, this effect is most pronounced at TADA2B binding sites (Fig. 4B and fig. S4D). Consistent with these observations, motifs at TADA2B-bound sites demonstrating a decrease in MYCN binding were strongly enriched for MYC/MYCN E-boxes (fig. S4E). In contrast with the global decrease in H3K9ac, we did not observe a statistically significant global alteration of H3K27ac after 6 hours of dTAG^V^-1 treatment (Fig. 4C and fig. S4F). However, a reduction of H3K27ac is observed at TADA2B binding sites 6 hours after dTAG^V^-1 treatment (Fig. 4C and fig. S4G).

### TADA2B loss leads to a down-regulation of MYCN targets and cell cycle–related genes

We were next interested in understanding the consequences of TADA2B loss on gene expression and whether the alterations in histone acetylation and MYCN binding that we observed after TADA2B loss lead to any change in gene expression. To that end, we performed RNA sequencing (RNA-seq) in our KELLY-TADA2B^deg^ model at 6, 24, and 72 hours after dTAG^V^-1 or DMSO treatment. To further confirm that the observed effects were the result of TADA2B loss, we also compared these expression signatures with constitutive CRISPR KO of *TADA2B* using two distinct sgRNAs 7 and 12 days after infection. We found that the two *TADA2B* sgRNAs showed high concordance with each other within each time point; we merged these data for downstream analyses (fig. S5, A and B).

**Fig. 5.**
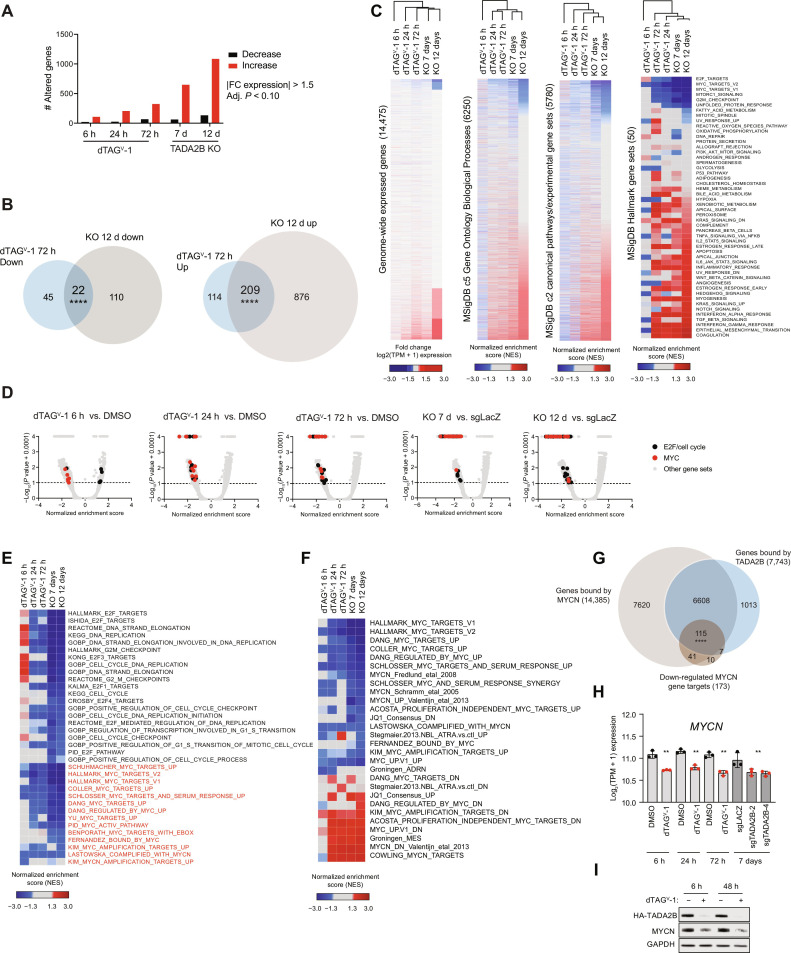
TADA2B loss leads to modest gene expression changes. (**A**) Bar plots depicting the number of differentially expressed genes induced by TADA2B loss (adjusted *P* ≤ 0.10, fold change expression ≥ 1.5). (**B**) Venn diagrams depicting the overlap between differentially expressed genes associated with TADA2B loss by CRISPR KO at 12 days and by degradation at 72 hours. Overlap significance, two-tailed Fisher’s exact test. ****P < 0.0001. (**C**) Left: Heatmaps showing gene expression alterations induced by TADA2B loss in dTAG^V^-1 and *TADA2B* KO experiments. Genes are ranked by expression changes induced by day 12 *TADA2B* KO. Center and right: Heatmaps for ssGSEA NES for the MSigDB C5, C2, and Hallmarks gene set collections. Hallmark gene set names are annotated. (**D**) Volcano plots for GSEA enrichment scores for the genome-wide expression changes induced by TADA2B loss in KELLY cells for the MSigDB gene set collection. Gene sets in overrepresented functional categories are highlighted. (**E**) Heatmap for ssGSEA NESs in the functional categories associated with TADA2B loss under dTAG^V^-1 versus DMSO and under sgTADA2B versus sgLACZ conditions. Gene set names are annotated. Color scale bars are shown at the bottom. (**F**) Heatmap for ssGSEA NESs for selected compendia of MYC, MYCN, and neuroblastoma-related gene set signatures associated with TADA2B loss under dTAG^V^-1 versus DMSO and under sgTADA2B versus sgLACZ conditions. (**G**) Venn diagrams depicting the overlap between down-regulated MYCN genes and binding of either MYCN or TADA2B as determined by ChIP-seq. Overlap significance, two-tailed Fisher’s exact test, ****P < 0.0001. (**H**) Bar plots depicting the *MYCN* expression changes induced by dTAG^V^-1 versus DMSO and sgTADA2B versus sgLACZ. **DESeq2 adjusted *P* ≤ 0.01. (**I**) Western blot depicting HA-TADA2B and MYCN protein levels in KELLY-TADA2B^deg^. GAPDH serves as a loading control.

At each time point, we found more genes with increased expression than decreased expression at permissive and restrictive significance thresholds (Fig. 5A and fig. S5C), which was unexpected given the reported role for the SAGA complex as a transcriptional coactivator. However, the genes and gene sets perturbed by TADA2B loss were notably consistent across perturbation modalities and time points although the gene expression consequences became more pronounced at later time points (Fig. 5, B and C, and fig. S5, D and E). Furthermore, in contrast with the substantial growth defect of SAGA complex loss and global loss of H3K9ac, we found that these gene expression changes were modest in magnitude even at 12 days (fig. S5F).

We noticed that the alterations in gene expression at all time points converged on several key functional groups of genes, including MYC signaling and E2F/cell cycle genes. When we considered these key functional groups over time, we found that the time course revealed interesting gene expression dynamics. Expression of the MYC-related gene sets began to decrease by 6 hours after dTAG^V^-1 treatment, and this down-regulation became more pronounced at later time points (Fig. 5, D and E, and fig. S5G). In contrast, the E2F/cell cycle–related gene sets were not significantly altered at 6 hours after dTAG^V^-1 treatment, but by 24 hours, these gene sets were down-regulated, a trend that continued at later time points (Fig. 5, D and E, and fig. S5H). Together, these data suggest that the down-regulation of MYC-related gene sets may be a more direct and immediate effect of SAGA complex loss, while E2F/cell cycle dysregulation might be a later consequence of this early loss of MYC related signaling. The observed decrease in MYC-related signaling and cell cycle–related gene sets is consistent with our data demonstrating that loss of TADA2B impairs cell cycle progression and alters MYCN binding to chromatin.

KAT2A, a KAT module member of the SAGA and ATAC complexes, has been reported to colocalize with MYC on chromatin and promote transcription in other settings (*27*–*29*), which is consistent with our finding that MYC target gene expression is impaired after TADA2B loss. Given that MYCN is closely related to MYC and is displaced from TADA2B binding sites after TADA2B loss, we were particularly interested in whether SAGA complex loss also affected the MYCN gene expression output. MYCN signatures are not well represented in the MSigDB compendium, so we compiled a set of MYCN gene sets described in the literature (table S3) and assessed whether these gene sets were altered after TADA2B loss. We found that MYCN gene signatures were decreased acutely, and this down-regulation was more pronounced at later time points (Fig. 5F). Further, we found that the large majority (~66%) of down-regulated genes are cobound by TADA2B and MYCN (Fig. 5G). These findings are consistent with the hypothesis that the KAT activity of the SAGA complex contributes to MYCN gene expression output.

Given that we had observed a decrease in MYCN binding after TADA2B loss, as well as a decrease in MYCN target gene expression, we next assessed whether *MYCN* expression itself was altered in our RNA-seq data. We found that there was a slight decrease in *MYCN* transcript levels after TADA2B loss at every time point (Fig. 5H). However, this is unlikely to explain the alterations observed in our ChIP data, as MYCN occupancy is reduced at TADA2B binding sites. Previous work has demonstrated that KAT2A can also directly acetylate and regulate MYC and MYCN protein stability (*30*), although it remains unclear whether this acetylation activity is affected when KAT2A is associated with the SAGA complex or the ATAC complex. We therefore assessed MYCN protein levels to determine whether there was a loss of protein stability induced by loss of TADA2B. We observed a decrease in MYCN protein levels, which was similar across time points (Fig. 5I). These data suggest that the SAGA complex may also play a role in maintaining MYCN protein stability and/or transcript levels.

### The histone acetyltransferases KAT2A and KAT2B are functionally redundant in neuroblastoma

**Fig. 6.**
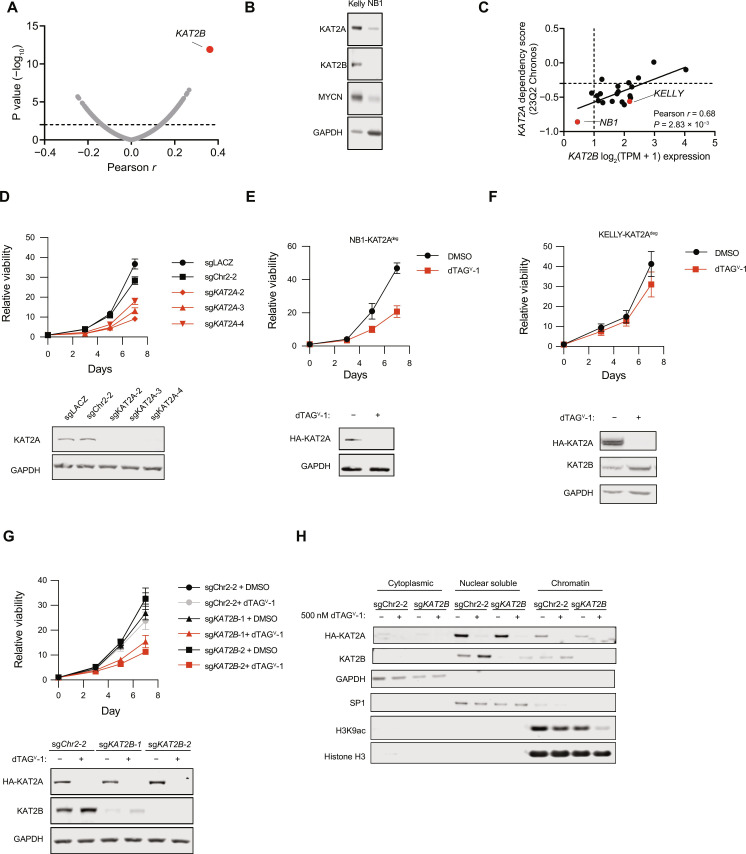
KAT2A and KAT2B are functionally redundant in neuroblastoma. (**A**) Volcano plot depicting the Pearson correlation between *KAT2A* dependency and gene expression across all *TADA2B*-dependent cell lines in the 23Q2 DepMap data. Each dot represents a single gene. Significant *t*-distribution cutoff *P* value of ≤0.01. (**B**) Western blot showing expression of KAT2A, KAT2B, and MYCN. GAPDH is shown as a loading control. (**C**) Scatter dot plot depicting the linear association between the *KAT2A* dependency gene effect and the *KAT2B* gene expression in neuroblastoma. Significance was determined by *F* test; cutoff *P* ≤ 0.01. (**D**) Relative viability is shown on the *y* axis for NB1 cells infected with sgRNAs targeting control (black) or *KAT2A* (red). (**E**) Relative viability NB1-KAT2A^deg^ cells treated with 1 μM dTAG^V^-1 or DMSO. Means + SD shown. Below, Western blot depicts exogenous KAT2A protein levels 24 hours after treatment with dTAG^V^-1 or DMSO as indicated. GAPDH is shown as a loading control. (**F**) KELLY cells, as in (E). Below, exogenous KAT2A and endogenous KAT2B protein levels are shown 24 hours after treatment with dTAG^V^-1 as indicated. GAPDH serves as a loading control. (**G**) Relative viability for KELLY-KAT2A^deg^ cells expressing sgRNAs targeting Chr2-2 or *KAT2B* and treated with DMSO or dTAG^V^-1 as indicated. Means + SD shown. Below, Western blot depicts HA-KAT2A and KAT2B levels in KELLY-KAT2A^deg^ with or without *KAT2B* KO and treated with DMSO or 500 nM dTAG^V^-1 for 24 hours. GAPDH serves as a loading control. (**H**) KELLY-KAT2A^deg^ cells were infected with sgRNAs targeting Chr2-2 (cutting control) or *KAT2B* as indicated and then treated with DMSO or dTAG^V^-1. A cell fractionation was performed, and exogenous KAT2A, endogenous KAT2B, and H3K9ac levels were assessed. GAPDH, SP1, and histone H3 serve as loading controls for the cytoplasmic, nuclear, and chromatin fractions, respectively.

*TADA2B* is unique to the SAGA complex and required for KAT activity, making it a useful tool to selectively perturb this activity. However, TADA2B itself is not readily druggable. TADA2B is in the KAT module and is required for efficient acetyltransferase activity (*31*–*33*), suggesting that the loss of KAT activity could be responsible for the phenotype we observed. KAT activity is more readily druggable. However, dependency on *KAT2A*, which encodes KAT2A (also commonly referred to as GCN5), the canonical catalytic KAT associated with SAGA, was not significantly different in *MYCN*-amplified neuroblastoma compared to other cell lines (fig. S1B). To understand this apparent discrepancy, we used *TADA2B* as a proxy for dependency on SAGA complex KAT activity and investigated genes whose expression is associated with *KAT2A* dependency in cell lines that depend on *TADA2B* [Computational correction of copy-number effect in CRISPR-Cas9 essentiality screens (CERES) score < −0.3] to determine why these cell lines were not uniformly dependent on *KAT2A*. The strongest correlation across these cell lines was low expression of *KAT2B*, a highly similar paralog of *KAT2A* (Fig. 6A). KAT2B, commonly known as P300/CBP-associated factor (PCAF), is also found in the SAGA complex in a mutually exclusive manner with KAT2A (*34*). While KAT2A and KAT2B have some nonredundant functions during development (*35*, *36*), the correlation between *KAT2A* dependency and *KAT2B* expression suggests that KAT2A and KAT2B might be functionally redundant in the SAGA complex in some cancers, including neuroblastoma (fig. S6A).

To interrogate the possibility that KAT2A and KAT2B are functionally redundant in this context, we used two *MYCN*-amplified neuroblastoma cell lines, KELLY and NB1, that depend on *TADA2B* but have high and low *KAT2B* expression, respectively (Fig. 6, B and C). NB1 has relatively low expression of *KAT2B* and is dependent on *KAT2A* (Fig. 6, C and D). We again used the FKBP12-based degron system for inducible protein degradation (*37*, *38*) and fused the cDNA of *KAT2A* to the FKBP12^F36V^ fragment and an HA tag (KAT2A^deg^) (fig. S6B). We generated neuroblastoma cell lines expressing this construct and knocked out endogenous *KAT2A* using CRISPR-Cas9 with an sgRNA that spans an intron/exon junction and therefore cannot cut the exogenous cDNA of *KAT2A*. We confirmed endogenous *KAT2A* editing using TIDE sequencing (fig. S6, C and D). In NB1, degradation of KAT2A had a substantial impact on viability (Fig. 6E). In contrast, in KELLY, which has comparatively higher *KAT2B* expression, degradation of KAT2A only modestly affected viability (Fig. 6F). Neither parental cell line is affected by the addition of dTAG^V^-1 alone (fig. S6, E and F). We also noted that protein levels of KAT2B were increased after KAT2A degradation in KELLY, consistent with a potential compensatory effect (Fig. 6F). We then knocked out *KAT2B* with two distinct sgRNAs and found that KELLY cells were now more sensitive to KAT2A degradation (Fig. 6G), indicative of functional redundancy between these two paralogs in neuroblastoma. In NB1, which already has low *KAT2B* expression, KO of *KAT2B* did not improve response (fig. S6G).

Further supporting this putative functional redundancy, we found that degradation of KAT2A alone was not sufficient to affect H3K9ac in KELLY cells but H3K9ac was reduced in the context of *KAT2B* KO and KAT2A degradation (Fig. 6H). Furthermore, we noted that KAT2B levels in the nuclear- and chromatin-bound fractions increased after KAT2A degradation, consistent with a model where KAT2B may replace KAT2A when KAT2A is lost. These data suggest that impairment of the histone acetyltransferase activity of SAGA affects cell viability, but because KAT2A and KAT2B are functionally redundant in this context, both KATs need to be impaired to block histone acetyltransferase activity. Thus, this redundancy may explain why *MYCN*-amplified neuroblastoma depends on *TADA2B*, which is required for full KAT activity.

### Pharmacologic degradation of KAT2A/KAT2B reduces neuroblastoma viability

**Fig. 7.**
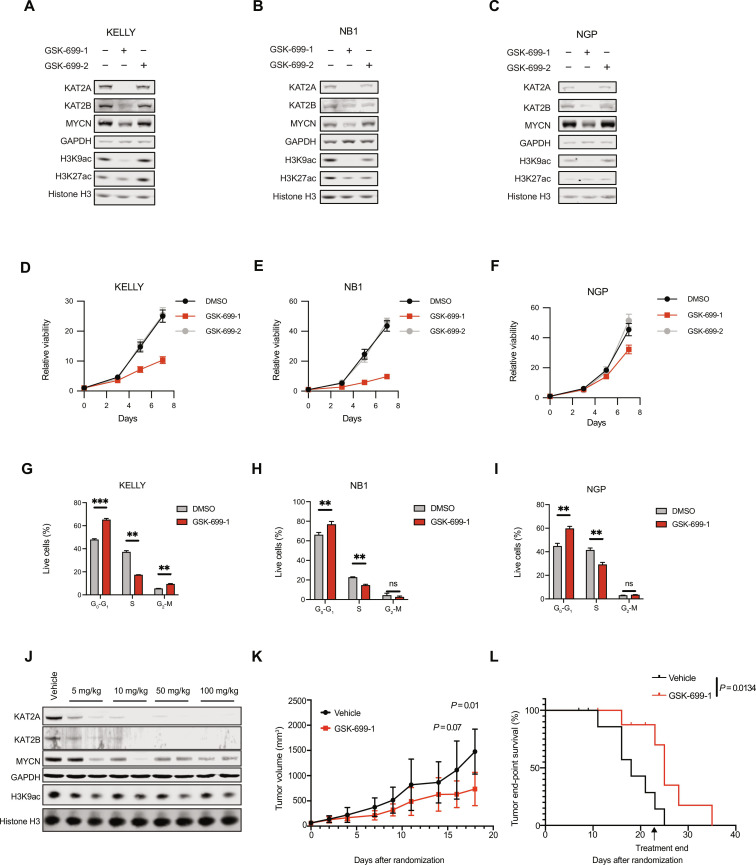
KAT2A and KAT2B degradation reduces neuroblastoma growth. (**A** to **C**) Western blot showing expression of KAT2A, KAT2B, MYCN, H3K9ac, or H3K27ac in neuroblastoma cell lines treated for 24 hours with vehicle, GSK-699-1, or GSK-699-2. GAPDH and histone H3 are shown as loading controls. (**D** to **F**) Relative viability for neuroblastoma cell lines treated with vehicle, 100 nM GSK-699-1, or 100 nM GSK-699-2. Means and SD are shown. (**G** to **I**) Neuroblastoma cell lines were plated and treated with DMSO or 100 nM GSK-699-1 for 72 hours, and then an EdU and PI incorporation assay was performed. The means and SD of three biological replicates are shown. Significance was determined with multiple unpaired *t* tests with Welch correction for grouped analysis. **P < 0.01 and ***P < 0.001. (**J**) Western blot showing expression of KAT2A, KAT2B, MYCN, or H3K9ac in KELLY xenograft tumors treated for 5 days with vehicle or GSK-699-1 (5, 10, 50, or 100 mg/kg) by intraperitoneal injection daily. (**K**) Tumor growth curves showing means + SD of tumor volume for tumors that received at least 18 days of vehicle (*n* = 5) or GSK-699-1 (50 mg/kg; *n* = 7) treatment. A two-sided Student’s *t* test determined significance at each time point. *P* values are indicated on days 16 and 18. (**L**) Kaplan-Meier survival curves showing end-point survival as determined by ≥20 mm in any direction or a maximal tumor burden of ~1800 mm^3.^ Eighteen mice were injected with KELLY cells into the subcutaneous flank and randomized into vehicle (*n* = 9, black) or GSK-699-1 (50 mg/kg ip daily; *n* = 9, red) cohorts. Mice that were euthanized before tumor end point due to ulceration are indicated with hashes on the curves (two for vehicle and three for GSK-699-1). Significance was determined by log-rank (Mantel-Cox) test.

No small-molecule inhibitors currently exist to inhibit the function of TADA2B. A proteolysis targeting chimeric (PROTAC) molecule that induces the proteolysis of both KAT2A and KAT2B proteins has been recently developed (*39*). On the basis of our previous data showing that simultaneous KAT2A/KAT2B loss reduced neuroblastoma viability, we hypothesized that the KAT2A/KAT2B PROTAC, GSK-699, may be a pharmacologic intervention for *MYCN*-amplified neuroblastoma. We confirmed across three *MYCN*-amplified cell line models that GSK-699-1 treatment, and not the related inactive enantiomer GSK-699-2, resulted in loss of both KAT2A and KAT2B expression and subsequent reduction of H3K9ac, consistent with loss of SAGA KAT activity (Fig. 7, A to C). Similar to TADA2B degradation, loss of KAT2A/KAT2B also resulted in decreased MYCN protein expression. GSK-699-1 is effective with low-nanomolar potency (fig. S7, A and B) and significantly reduced neuroblastoma growth over prolonged treatment (Fig. 7, D to F). In addition, we treated three non–*MYCN*-amplified cell lines with GSK-699-1 and observed heterogeneous responses similar to observations with *TADA2B* KO (figs. S2B and S7C). Sensitivity to GSK-699-1 in the non–*MYCN*-amplified models was associated with a significantly reduced MYC expression (fig. S7D). Reduced growth with GSK-669-1 in *MYCN*-amplified models was predominantly through G_1_-G_0_ cell cycle arrest (Fig. 7, G to I). Together, these data suggest that therapeutic targeting of the SAGA complex via degradation of KAT2A and KAT2B may be a therapeutic strategy for *MYCN*-amplified neuroblastoma.

While KAT2A/KAT2B degradation showed promising activity as a therapeutic approach in cell culture models, it is important to establish the therapeutic potential of pharmacologic agents in animal models. To evaluate whether GSK-699-1 is effective in vivo, we first performed a dose-finding study with KELLY neuroblastoma cells injected into the subcutaneous tissue of NOD scid gamma (NSG) mice and treated with increasing doses of GSK-699-1. We observed consistent KAT2A/KAT2B degradation with doses as low as 10 mg/kg injected intraperitoneally (ip) once daily and up to 100 mg/kg ip daily (Fig. 7J). As we observed in cell culture models, degradation of KAT2A/KAT2B was associated with reduced MYCN protein expression and reduced H3K9ac as compared to the vehicle control. Although notably, H3K9ac levels are only reduced by ~50% as compared to the vehicle, suggesting that SAGA activity may not be completely ablated. We moved forward with 50 mg/kg once daily dosing for 21 days as we had observed adequate degradation, no adverse effects, and good solubility of the compound.

To assess the efficacy of GSK-699-1 on tumor growth, we randomized NSG mice harboring KELLY xenografts to receive either vehicle or injection of GSK-699-1 (50 mg/kg once daily ip) for 21 days. GSK-699-1 treatment significantly slowed tumor progression and increased end-point survival (Fig. 7, K and L, and fig. S7E). Furthermore, the treatment was well tolerated as assessed by mouse weights (fig. S7F). We continued to observe decreased KAT2A/KAT2B expression at tumor end point for mice receiving GSK-699-1 (fig. S7G). Together, these data show that GSK-699-1 is effective at on-target degradation and efficacy in animal models.

### Epigenetic and transcriptional alterations following KAT2A/KAT2B degradation correlate with loss of TADA2B

We next sought to correlate the transcriptional and epigenetic effects of KAT2A/KAT2B degradation with the previously generated TADA2B epigenetic and transcriptional data to assess whether KAT2A/KAT2B degradation results in the loss of SAGA activity. To directly compare phenotypic effects with the previously generated TADA2B degradation data, we performed calibrated ChIP-seq for H3K9ac, H3K27ac, and MYCN at 6 hours in addition to RNA-seq at 6, 24, and 72 hours.

**Fig. 8.**
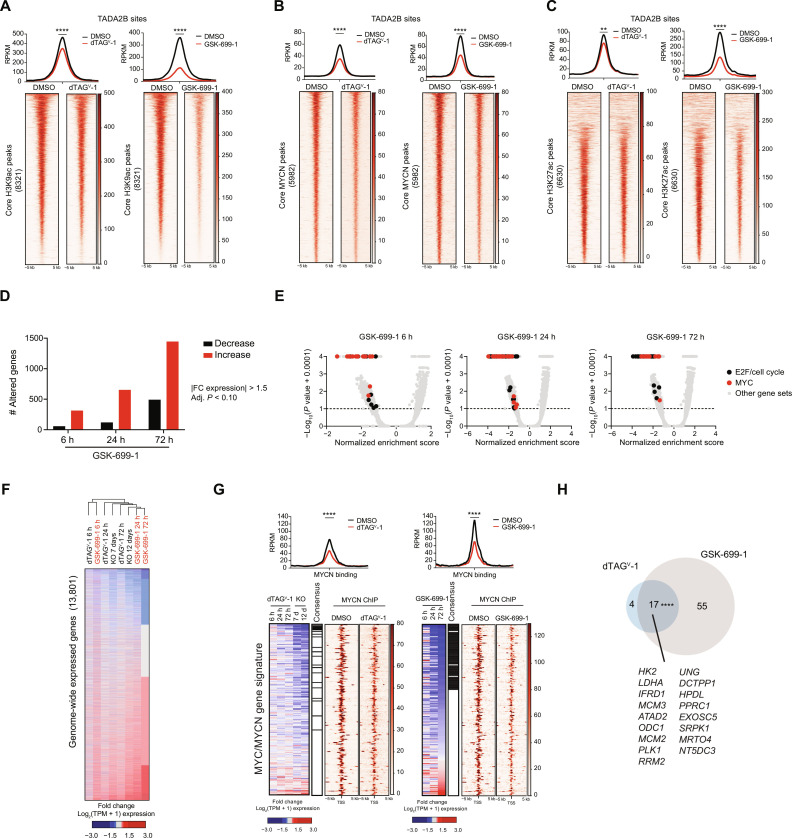
Integrated sequencing reveals regulation of SAGA-regulated genes with GSK-699-1. (**A** to **C**) Heatmaps of H3K9ac (A), MYCN (B), or H3K27ac (C) restricted to TADA2B-bound sites in either KELLY-TADA2B^deg^ cells treated with DMSO or 500 nM dTAG^V^-1 for 6 hours (left) or KELLY cells treated with DMSO or 100 nM GSK-699-1 for 6 hours (right). The regions are ranked on the basis of the binding signal in DMSO. At top, read density metaplots showing average RPKM-normalized signal for antibodies across the universe of binding sites depicted below. Differential read density between conditions estimated on the basis of unpaired *t* test with Welch’s correction. (**D**) Bar plot depicting the number of differentially expressed genes induced by GSK-699-1 treatment (DESeq2 adjusted *P* ≤ 0.10, |fold change expression| ≥ 1.5). (**E**) Volcano plots for GSEA enrichment scores for the genome-wide expression changes induced by GSK-699-1 treatment in KELLY cells. (**F**) Heatmap for log_2_ fold change in gene expression across RNA-seq generated for KELLY-TADA2B^deg^ and GSK-699-1 datasets. Heatmap is sorted on the basis of the GSK-699-1 72-hour time point and organized on the basis of hierarchical clustering. (**G**) Integrated heatmap showing RNA expression across experiments and the corresponding enrichment for MYCN by ChIP-seq in vehicle-treated, dTAG^V^-1–treated (left), or GSK-699-1–treated (right) cells for the MYC/MYCN-related gene signatures. Black bars indicate consensus genes for which there was down-regulation of the gene by RNA-seq and loss of MYCN binding by ChIP-seq. Above, read density metaplots showing average RPKM-normalized signal for MYCN across the universe of binding sites depicted below. Differential read density between conditions estimated on the basis of unpaired *t* test with Welch’s correction. (**H**) Venn diagram showing the number of unique and overlapping consensus genes as determined for each treatment in (G). Overlapping genes are indicated. Significance determined by two-tailed Fisher’s exact test, ****P < 0.0001.

Six hours following GSK-699-1 treatment, we observed a reduction of H3K9ac deposition genome-wide and at the previously identified TADA2B-bound sites (fig. S8A). Similarly, there was decreased genome-wide MYCN binding with a slightly higher proportion of decreased MYCN binding at TADA2B-bound sites (fig. S8B). In contrast to TADA2B degradation, KAT2A/KAT2B degradation resulted in decreased global H3K27ac occupancy that was more notable at TADA2B sites (fig. S8, C and D). We did not see degradation of the H3K27 acetyltransferase P300 (fig. S8E). Thus, this discrepancy may be due to changes in the activity of CBP/P300 as KAT2B/PCAF is known to interact and augment their activity. To directly compare epigenetic effects between KAT2A/KAT2B degradation and TADA2B degradation, we analyzed core binding sites that were shared between both datasets (fig. S8F). Significant decreases in H3K9ac and MYCN occupancy were noted for KAT2A/KAT2B degradation and TADA2B^deg^ datasets, particularly at TADA2B-specific sites (Fig. 8, A to C). Genome-wide, H3K9ac, H3K27ac, and MYCN are more strongly reduced with GSK-699-1 relative to TADA2B degradation (fig. S8, G to I). This discrepancy is likely due to combined KAT2A and KAT2B degradation perturbing the function of both SAGA and ATAC complexes. However, the epigenetic effects of KAT2A/KAT2B degradation correlate with the loss of SAGA complex activity as determined by genetic loss of TADA2B, suggesting that loss of SAGA activity is one of the relevant effects of GSK-699-1 treatment.

We next evaluated transcriptional changes that occurred with KAT2A/KAT2B degradation. RNA-seq analysis with GSK-699-1 treatment showed significant alterations in gene expression that increased in magnitude with time (Fig. 8D). Similar to RNA-seq with TADA2B KO or degradation, there was a bias toward gene up-regulation with KAT2A/KAT2B degradation (Figs. 5A and 8D). However, the magnitude and number of genes that increased are larger, again likely because of perturbation of both the SAGA and ATAC complexes. Similarly, GSEA showed that down-regulated genes are enriched for MYC, E2F/cell cycle, and MYCN-related gene signatures across all three time points (Fig. 8E and fig. S9A). Intersection of RNA-seq between GSK-699-1 and TADA2B^deg^ datasets demonstrated a strong correlation of gene expression changes induced by GSK-699-1 as compared to TADA2B degradation and *TADA2B* KO at all of the observed time points (Fig. 8F and fig. S9B). Notably, the magnitude and number of gene expression changes induced by GSK-699-1 were greater than either TADA2B degradation or KO alone (fig. S9, C and D). These data support that GSK-699-1 treatment strongly correlates with epigenetic and transcriptional signatures observed with TADA2B loss of function.

We have consistently observed that MYCN and E2F/cell cycle gene expression signatures are decreased across all treatments and time points (fig. S9E). We hypothesized that this is likely due to decreased MYCN binding at target genes, thereby resulting in their decreased expression. To evaluate this, we compared transcriptional and epigenetic alterations specifically at genes found within the MYC/N and E2F gene sets following either TADA2B degradation or GSK-699-1 treatment. We observed that MYCN binding is significantly reduced in both TADA2B^deg^ and GSK-699-1 experiments for both MYCN- and E2F-related gene sets (Fig. 8G and fig. S9F). We identified 21 and 72 consensus genes that lose MYCN binding and result in decreased gene expression in TADA2B^deg^ and GSK-699-1, respectively (Fig. 8H and table S4). Of these, 17 genes are shared between the two treatments and encode many proteins known to be involved in cell cycle such as Polo-like Kinase 1 (PLK1), Minichromosome Maintenance Complex Component 3 (MCM3), and Minichromosome Maintenance Complex Component 2 (MCM2). For the E2F gene set, 19 and 79 genes demonstrated decreased MYCN binding and decreased gene expression in TADA2B^deg^ and GSK-699-1, respectively (fig. S9G and table S4). Of the 18 overlapping genes, many of the same cell cycle genes were shared. Together, these data suggest that SAGA loss of function can reduce cell cycle–related signatures through reduction of MYCN levels and altered binding on chromatin and suggest a potential mechanism to target *MYCN*-amplified neuroblastomas.

## DISCUSSION

Genome-scale CRISPR-Cas9 dependency datasets have allowed the identification of many single-gene dependencies in cancer that may be promising new therapeutic targets (*24*, *40*–*42*). Here, instead of focusing on single-gene outlier dependencies, we use CRISPR screening datasets to identify a convergence of selective dependencies on a single epigenetic complex, an approach that has also been proposed by others (*43*). We were further able to focus our validation efforts on the key relevant subunits of the SAGA complex through analyzing these dependency datasets. Using this approach, we identify and describe a role for the SAGA complex acetyltransferase activity in maintaining the oncogenic state in *MYCN*-amplified neuroblastoma. Histone-modifying complexes play an essential cooperating role with oncogenic gene expression programs, and, hence, they represent an exciting class of targets in transcriptionally driven pediatric cancers that lack directly targetable oncogenes. We demonstrate that CRISPR screen data can be effectively used to uncover complex level dependencies and focus efforts on key functional subunits, an approach that could be readily applied to other cancer lineages and subtypes.

We find that loss of SAGA complex KAT activity in *MYCN*-amplified neuroblastoma, through modulation of TADA2B, leads to a rapid and global loss of acetylation on H3K9, including in the promoters of actively transcribed genes. However, we do not observe similarly widespread gene expression changes. Instead, we observe a relatively modest alteration to gene expression, consistent with previous findings that the SAGA complex is not required for global gene expression (*26*). Here, we find many genes that lose H3K9ac in their promoter after TADA2B degradation but experience no change in gene expression or have an increase in gene expression despite loss of this epigenetic mark. This observation underscores that the relationship between individual histone marks and gene expression is complex and that loss of an individual mark may not be sufficient at most gene sites to affect expression as there are many other histone-modifying complexes and histone marks that might ensure some level of redundancy. In addition, it is possible that the primary function of H3K9ac is not to promote gene expression and that global loss of this mark could be affecting cell viability through nontranscriptional means that we do not capture in the present study.

We focused our efforts on understanding the function of SAGA-mediated histone acetylation in the context of *MYCN*-amplification because this is a disease area of particular clinical need. However, we note that some non–*MYCN*-amplified neuroblastoma cell lines are as dependent on SAGA activity as *MYCN*-amplified cell lines. Low-throughput validation showed that in at least two non–*MYCN*-amplified cell lines, a dependency on SAGA exists. This is relevant as non–*MYCN*-amplified lines typically express MYC and treatment with GSK-699-1 demonstrated a reduction in expression of MYC. Thus, non–*MYCN*-amplified lines may also depend on SAGA activity through a similar MYC-related mechanism. However, not all cell lines overexpressing *MYC* showed impaired growth with *TADA2B* KO or KAT2A/KAT2B degradation, suggesting that there might be a specific context that underlies sensitivity to SAGA loss of function. Further work will be needed to assess the potential utility of SAGA loss of function in the non–*MYCN*-amplified context. As these dependency datasets continue to grow, with an emphasis on expanding the representation of nonamplified neuroblastoma lines, we should be able to determine whether this vulnerability is unique or more pronounced in *MYCN*-amplified compared to *MYCN*–wild-type neuroblastoma.

We find that SAGA complex colocalizes with MYCN, consistent with previous reports that KAT2A is recruited to MYC binding sites and that MYCN and KAT2A regulate overlapping genes in neural stem cells (*21*, *27*, *28*). We note, however, that these previous studies focused on KAT2A and do not account for either functional redundancy between KAT2A and KAT2B or distinct functions of the SAGA and ATAC complexes. Further, while our system shows SAGA-specific localization using TADA2B as a proxy, overexpression of *TADA2B* using the dTAG system may not perfectly represent localization of the endogenous complex. Future studies evaluating the localization of endogenous TADA2B will be important to confirm our findings. Unexpectedly, our data support that TADA2B degradation leads to a rapid loss of MYCN from chromatin, particularly at sites identified as TADA2B binding sites, suggesting that MYCN localization is strongly dependent on SAGA activity. Acute loss of MYCN is associated with loss of MYCN-driven gene expression, and this decrease in gene expression is sustained at later time points. Further, the impact of pharmacologic degradation of KAT2A/KAT2B strongly correlated with the impact of TADA2B degradation, supporting the importance of the SAGA complex in maintaining *MYCN*-amplified neuroblastoma.

A key remaining question is why the degradation of TADA2B leads to rapid displacement of MYCN at TADA2B binding sites. KAT2A acetylation activity has been shown to be a critical regulator of MYC and MYCN protein stability (*30*). This might be relevant in the current study as transcriptional alterations were not well correlated with global H3K9ac and H3K27ac. Further, acetylation-independent activation of gene regulation by SAGA has been demonstrated, and our studies do not rule out an acetyltransferase-independent SAGA function in neuroblastoma, perhaps due to interactions with transcription factors such as MYCN (*44*, *45*). In addition, this study focused on KAT2A activity without interrogating potential differences between KAT2A activity in the SAGA and ATAC complexes. KAT2A has been shown to have distinct functions when incorporated into SAGA or ATAC during hematopoiesis (*46*), and we observe very different dependency profiles for *TADA2A* (ATAC) and *TADA2B* (SAGA), suggesting that some functions may be complex specific. Therefore, revisiting the role of KAT2A for MYC and MYCN stability with a focus on SAGA versus ATAC acetyltransferase activity might be very informative. Prior studies have interrogated the function of KAT2A in MYC-driven cancer (*47*, *48*). These studies, however, largely focused on KAT2A and did not interrogate redundant functions of KAT2A and KAT2B. In the context of B cell lymphoma, however, loss of KAT2A activity alone had a significant impact on disease progression in a mouse model (*47*). Although our findings cannot be directly compared to prior work in MYC-driven cancers, our studies clearly show that perturbing TADA2B impairs SAGA complex KAT activity regardless of which KAT is associated.

Here, we identify that members of the core and KAT modules drive the selective dependency on the SAGA complex in *MYCN*-amplified neuroblastoma. We report that the acetyltransferase activity of the SAGA complex is required to maintain the oncogenic state in neuroblastoma and that impairing this activity either through TADA2B KO/degradation or through combined loss of KAT2A/KAT2B impairs neuroblastoma cell growth. This suggests that small-molecule targeting of this complex could have therapeutic relevance for this disease. We find that KAT2A and KAT2B have functionally redundant roles in SAGA, so inhibition or degradation of both would likely be required. Histone acetyltransferase inhibitors, bromodomain inhibitors, and a PROTAC degrader molecule that targets both KAT2A and KAT2B have been reported (*39*, *49*, *50*). On the basis of our findings using the PROTAC degrader GSK-699-1, combined loss of KAT2A/KAT2B reduced neuroblastoma proliferation and induced transcriptional and epigenetic changes that are consistent with TADA2B degradation. Thus, the cell viability effect is likely mediated through the SAGA complex, although we cannot rule out that the ATAC complex may facilitate some of the phenotypes observed with KAT2A/KAT2B degradation. Further, GSK-699-1 showed robust degrader activity in vivo and significantly slowed neuroblastoma tumor progression. However, tumors eventually overcome KAT2A/KAT2B degradation, suggesting that more complete KATA2A/KAT2B degradation may be necessary for tumor regression or that SAGA inhibition may require combinatorial therapies.

We report a role for the SAGA complex acetyltransferase activity in maintaining the oncogenic state in *MYCN*-amplified neuroblastoma, expanding the universe of histone-modifying complexes that are central to maintaining the disease state. Once we have a complete understanding of the histone modifiers that contribute to neuroblastoma oncogenesis, it will be important to interrogate how the activity of these different complexes may antagonize, complement, or regulate each other’s activity and localization to maximally exploit these epigenetic vulnerabilities in this clinically challenging disease.

## MATERIALS AND METHODS

### Cell lines

All neuroblastoma cell lines were collected by the Cancer Cell Line Encyclopedia and DepMap projects. The sources for these lines are listed at depmap.org, and they can be obtained from their respective sources. Their identities were confirmed by single-nucleotide polymorphism array and by short tandem repeats (STR) profiling (Labcorp). All cell lines were confirmed negative for mycoplasma infection (Lonza MycoAlert). KELLY, SH-SY5Y, CHLA-255, NB69, TC32, and NB1 were cultured in RPMI 1640 supplemented with 10% fetal bovine serum. NGP, U20S, and EW8 were cultured in Dulbecco’s modified Eagle’s medium supplemented with 10% fetal bovine serum.

### Single-guide RNAs

For *KAT2B* KO studies, we used a hygromycin selectable vector lenti-sgRNA hygro (Addgene, #104991) with the indicated sgRNA (sg*Chr2-2* or sg*KAT2B*). Lenti-sgRNA hygro was a gift from B. Stringer (Addgene, plasmid #104991; http://n2t.net/addgene:104991; RRID:Addgene_104991) (*51*). For all other experiments, the sgRNA was cloned into lentiCRISPR v2 containing Cas9 and either puromycin or blasticidin resistance markers. LentiCRISPR v2 was a gift from F. Zhang (Addgene, plasmid #52961; http://n2t.net/addgene:52961; RRID:Addgene_52961) (*52*). For low-throughput experiments, the following guide sequences were used: sgTAD2AB-2, TCAGCCACAGAGATGTCCAG; sgTADA2B-4, GACAGGTGTGGTCTGTCACG; sgTADA2B-5, GCTGGTAGCCGTG-GTAGCGG; sgTADA2B-6, GGGCGGCTGGACCAGTCGCG; sgChr2-2, GGTGTGCGTATGAAGCAGTG; sgPOLR1C, AAGAATCTCATCCTGAACAA; sgKAT2A-2, GCGCTGGAAAAGTTCCGAG; sgKAT2A-4, TTCTCAAGCTTCTTGGCGCG; sgKAT2B-1, GCAGGCGCCCGAGCCCCCGG; sgKAT2B-3, GCTCCTGCCCCCGCAGCCGCC; sgTAF5L-1, AAACAGTCCGAAGAGCACAG; sgTAF5L-2, GCAGAAACATTCCATGGAAG.CRISPR editing was validated using TIDE sequencing (TADA2B only) and Western blotting.

### sgRNA viability assays

For low-throughput viability assays, cells were transfected with a vector containing Cas9 and the indicated sgRNA as described above and 7 days after infection seeded into 384-well plates or 96-well plates. For dTAG^V^-1 treatment, medium and compound were replenished on days 3, 5, and 7. CellTiter-Glo was used according to the manufacturer’s instructions to assess the relative viability at days 3, 5, and 7, normalized to day 0. Luminescence was determined using an EnVision plate reader. Experiments were done in triplicate, and technical replicates in each experiment were at least triplicate. On-target editing of the CRISPR guides was confirmed by TIDE sequencing or Western blot.

### Degrader viability assays

GSK-699-1 and GSK-699-2 were purchased from WuXi. For viability assays, ~5000 cells per well were seeded into 384-well plates. Cells were treated with either increasing concentrations of GSK-699-1 or GSK-699-2. CellTiter-Glo was used according to the manufacturer’s instructions to assess the relative viability at days 3, 5, and 7, normalized to day 0. Luminescence was determined using an EnVision plate reader. Experiments were plated in triplicate and with at least six technical replicates for each experiment.

### Exogenous expression of *TADA2B*

To render exogenous TADA2B resistant to sgTADA2B-4, the *TADA2B* open reading frame (ORF) was codon-optimized (G to A at position 390). This *TADA2B* sequence was cloned into the pLEX-305-C-dTAG vector. pLEX_305-C-dTAG was a gift from J. Bradner and B. Nabet (Addgene, plasmid #91798; http://n2t.net/addgene:91798; RRID:Addgene_91798) (*37*). To generate the TADA2B^deg^ lines with KO of endogenous *TADA2B* and degron-tagged exogenous TADA2B, cell lines were coinfected with a puromycin-resistant vector containing a codon-optimized *TADA2B* with a C-terminal FKBP12^F36V^-2× HA tag and a blasticidin-resistant vector containing an sgRNA-targeting *TADA2B* (sgTADA2B-4). Cell lines were selected with both blasticidin and puromycin.

### Exogenous expression of *KAT2A*

The *KAT2A* ORF was cloned out of the green fluorescent protein (GFP)–GCN5 plasmid. GFP-GCN5 was a gift from K. Miller (Addgene, plasmid # 65386; http://n2t.net/addgene:65386; RRID:Addgene_65386) (*53*). This ORF was cloned into the pLEX-305-C-dTAG vector (Addgene, #91798) using the Gateway Cloning system. To generate the KAT2A^deg^ lines with KO of endogenous *KAT2A* and degron-tagged exogenous *KAT2A*, cell lines were coinfected with a puromycin-resistant vector containing a *KAT2A* ORF with a C-terminal FKBP12^F36V^-2× HA tag and a blasticidin-resistant vector containing an sgRNA-targeting *KAT2A* (sgKAT2A-2). Because sgKAT2A-2 is in an intronic region, the exogenous *KAT2A* ORF was resistant to cutting. Resistant cell lines were selected with both blasticidin and puromycin.

### In vivo experiments

Our research studies comply with ethical guidelines determined by the Dana-Farber Cancer Institute Animal Care and Use Committee under protocol number 15-029.

### GSK-699-1 dose-finding study

KELLY cells were expanded as described above. A total of 3 × 10^6^ cells were resuspended in cell culture medium and mixed in a 70:30 medium:Matrigel (Corning) suspension before being injected unilaterally into the subcutaneous space of 22 NSG mice. Tumor size was determined three times weekly by digital caliper, and mouse weight was determined three times weekly. Once tumors were established (~50 to 100 mm^3^), mice were randomized into GSK-699-1 (5, 10, 50, or 100 mg/kg) or vehicle dosed by intraperitoneal injection each day. GSK-699-1 powder was resuspended in 10% DMSO in sterile phosphate-buffered saline (PBS). Mice were treated for 4 days and then euthanized 6 hours after the last dose for downstream analyses.

### GSK-699-1 efficacy study

KELLY cells were expanded as described above. A total of 3 × 10^6^ cells were resuspended in cell culture medium and mixed in a 70:30 medium:Matrigel (Corning) suspension before being injected unilaterally into the subcutaneous space of 18 NSG mice. Tumor size was determined three times weekly by digital caliper, and mouse weight was determined three times weekly. Once tumors were established (~50 to 100 mm^3^), mice were randomized into either GSK-699-1 (50 mg/kg) or vehicle dosed by intraperitoneal injection each day. GSK-699-1 powder was resuspended in 10% DMSO in sterile PBS. Experimental end point was determined by a tumor diameter of ≥20 mm in any direction or a maximal tumor burden of ~1800 mm^3^ or whether mice appeared to be unhealthy.

### Western blotting

For Figs. 2E, 5I, 6 (B and D to H), and 7 (A to C and J) and figs. S2 (E and F), S6G, S7G, and S8E, cells were lysed in Cell Signaling Technology cell lysis buffer (9803) supplemented with protease (Roche, #11836170001) and phosphatase inhibitors (Roche, #04906845001). Lysates were quantified using a Bicinchoninic acid assay (Pierce) and normalized. For Fig. 2 (A and D) and figs. S2 (A and B), S3B, and S7D, cells were lysed in 2× Laemmli sample buffer supplemented with 1× reducing agent. SDS–polyacrylamide gel electrophoresis gels were used to separate proteins, and proteins were transferred to a polyvinylidene difluoride membrane. Primary antibodies used in this study were as follows: glyceraldehyde-3-phosphate dehydrogenase (GAPDH) (Santa Cruz Biotechnology, #sc-47724), HA-tag (Cell Signaling Technology, #3724), TADA2B (Proteintech, #17367-1-AP), TAF5L (Proteintech, #19274-1-AP), HA-tag (Cell Signaling Technology, catalog no. 2367), KAT2B (Santa Cruz Biotechnology, #sc-13124; or Cell Signaling Technology, #3378), GCN5L2 (Cell Signaling Technology, #3305), acetyl–histone H3K9 (Cell Signaling Technology, #9649), SP1 (Cell Signaling Technology, #5931), MYCN (Santa Cruz Biotechnology, #SC-53993), and total H3 (Cell Signaling Technology, #3638 and #4499). Membranes were incubated with secondary antibodies (LI-COR, #926-68070 and #926-32211) and imaged on a LI-COR Odyssey.

### Antibodies for ChIP-seq

Antibodies used for ChIP were HA-tag (Cell Signaling Technology, #3724), H3K9ac (Abcam #ab4441), H3K27ac (Abcam #ab4729), and MYCN (Abcam #ab16898). For HA-tag ChIP in KELLY, 5 μg of HA antibody was used for 20 million cells. For NGP and NB1 HA-tag ChIPs, 50 μl of HA antibody was used for 20 million cells. For MYCN, 10 μg of antibody was used per sample of 20 million cells. For H3K9ac, 5 μg of antibody was used per sample of 5 million cells. For H3K9ac, 7.5 μl of spike-in chromatin was used per sample. For MYCN, 3 μl of spike-in chromatin was used per sample. For HA ChIP, 10 μl of spike-in chromatin was used per sample of 20 million cells. For H3K27ac, 5 μg of antibody and 2 μl of spike-in chromatin were used for 10 million cells.

For each sample, protein A or protein G beads (Life Technologies, #10003D) were prepared by washing three times in bovine serum albumin (BSA) blocking solution and then resuspended in 250 μl per sample of BSA blocking solution with appropriate antibody and spike-in antibody. Beads were incubated overnight with rotation, washed four times with BSA blocking solution, and resuspended in 100 μl per sample of BSA blocking solution. Spike-in chromatin was added to the beads 2 hours before adding sheared human chromatin and incubated with rotation at 4°C.

### Chromatin immunoprecipitation sequencing

Neuroblastoma cell lines were grown in 15-cm plates. A total of 20 million cells were cross-linked with 1% formaldehyde in 22 ml of warm medium for 10 min with rotation, followed by quenching with the addition of 1.1 ml of 2.5 M glycine for 5 min. Cells were pelleted by centrifugation at 600*g* and resuspended in 1 ml of cold PBS supplemented with protease inhibitors (Thermo Fisher Scientific, # 78430) and 1 mM phenylmethylsulfonyl fluoride (Sigma-Aldrich, #P7626). This wash was repeated twice for a total of three washes in PBS. Cells were resuspended in cytoplasmic and then nuclear lysis buffer, and cells were sheared on an E220 Covaris sonicator (duty cycle, 5%; peak power, 140 W; cycles per burst, 200; temperature, 4°C; time, 15 min). Sheared lysates were cleared by centrifugation at 15,000*g* for 10 min at 4°C and incubated overnight with rotation at 4°C with magnetic beads prebound with antibody, spike-in antibody (Active Motif, #61686), and spike-in chromatin (Active Motif, #53083). Following overnight incubation, beads were washed twice with low-salt wash buffer [0.1% SDS, 1% Triton X-100, 2 mM EDTA, 20 mM tris-HCl (pH 8.1), and 150 mM NaCl], twice with high-salt wash buffer [0.1% SDS, 1% Triton X-100, 2 mM EDTA, 20 mM tris-HCl (pH 8.1), and 500 mM NaCl], twice with LiCl wash buffer [0.25 M LiCl, 1% IGEPAL CA-630, 1% deoxycholic acid, 10 mM tris-HCl (pH 8.1), and 1 mM EDTA], and once with Tris-EDTA (TE) buffer. DNA was eluted in elution buffer (1% SDS and 0.1 M NaHCO_3_). Cross-linking was reversed overnight at 65°C with 0.9% ribonuclease A, 0.9% Proteinase K, and 0.182 M NaCl. DNA was isolated by adding Agencourt AMPure XP beads (Beckman Coulter, A63880) at 1.2× volume, incubating for 20 min, washing twice with 80% ethanol, and eluting into 10 to 20 μl of TE.

### Cell cycle analysis

Cell cycle analysis was performed using the Click-iT EdU Alexa Fluor 647 Flow Cytometry Assay Kit (Thermo Fisher Scientific, #C10419). Cells were grown in 10-cm plates or six-well plates and incubated with 10 μM EdU for 90 min. After harvesting, cells were washed with 3 ml of 1% BSA in PBS. Cell pellets were fixed and stained per the manufacturer’s protocol. Following ribonuclease A digestion and PI staining, samples were analyzed by flow cytometry using BD FACSDiva9.0.

### RNA sequencing

Cells were treated with DMSO or 500 nM dTAG^V^-1 as described or infected with sgRNAs targeting LACZ and TADA2B as described for the 7-day time point. KELLY cells were treated with DMSO or 100 nM GSK-699-1 for 6, 24, or 72 hours. Total RNA was collected from biological triplicates, homogenized by QIAshredder (QIAGEN, #79656) and purified using the RNeasy PLUS Mini Kit from QIAGEN (#74134). Preparation of RNA library and transcriptome sequencing was performed by Novogene Co. Ltd. For total RNA-seq of KELLY cells with sgRNAs at 12 days, libraries were made using Illumina TruSeq Stranded Total RNA Library Prep and sequenced using 2× 75-nucleotide paired-end protocol on Illumina HiSeq3000 instrument at MD Anderson Science Park Next-Generation Sequencing Facility.

### ATAC sequencing

ATAC-seq was performed as previously described with modifications (*54*, *55*). KELLY dTAG TADA2B cells were cultured under standard conditions and then treated with DMSO or 500 nM dTAG^V^-1 for 48 hours. The Tn5 transposition reaction was performed using the Illumina Tagment DNA TDE1 Enzyme and Buffer Kit (Illumina, #20034197). Cells were trypsinized, 100,000 collected, and washed once with cold PBS. A resuspension buffer was prepared [10 nM tris-HCl (pH 7.5), 10 mM NaCl, and 3 mM MgCl_2_]. Cells were lysed in 50 μl of lysis buffer (0.1% NP-40, 0.1% Tween 20, and 0.01% digitonin in resuspension buffer) for 3 min on ice and then washed with 1 ml of wash buffer (0.1% Tween 20 in resuspension buffer). Lysates were cleared by centrifugation at 500*g* for 10 min at 4°C. Tn5 transposition reaction was performed with the Nextera kit (Illumina). The nuclear pellet was resuspended in transposition reaction mix (25 μl of Tagment DNA Buffer, 16.5 μl of PBS, 0.5 μl of 10% Tween 20, 0.5 μl of 1% digitonin, 2.5 μl of Tagment DNA Enzyme 1, and 5 μl of nuclease-free water) and incubated for 30 min at 37C on a thermomixer with 1000 rpm. For KELLY cells with sgRNAs at 12 days, nucleus isolation and transposition reaction procedures were same as above except 100 μl of lysis buffer and 100 μl of transposition reaction mix were used for 100,000 cells. DNA was isolated using the QIAGEN MinElute Reaction Cleanup Kit (QIAGEN #28204) and eluted in 10 μl of elution buffer (EB). Library generation was performed with NEBNext High-Fidelity 2× PCR (polymerase chain reaction) Master Mix (New England Biolabs, #M0541S), SYBR Green I (Thermo Fisher Scientific, #S7563), Ad1_noMX and Ad2.* primers at 25 μM.

### Dependency data analysis

The CRISPR (DepMap Public 23Q2 + Score, Chronos) data from the screens published by Broad’s Achilles and Sanger’s SCORE projects (*56*) were downloaded from the DepMap portal https://depmap.org/portal/download/. The CRISPR 23Q2 screening was performed for 28 tumor lineages on 1095 cell lines, of which 37 are annotated as neuroblastoma. The gene effect scores summarizing the guide depletion were determined on the basis of the Chronos algorithm (*57*). Negative Chronos dependency scores below −0.3 estimate cell growth inhibition and/or death following gene KO. Common essential genes have a median Chronos score of −1.

The *MYCN* status for the NBL cell lines was estimated on the basis of the *MYCN* copy number and expression data available for 37 NBL cell lines in the 23Q2 DepMap repository and on the basis of *MYCN* status validation in relevant published studies (*58*, *59*). Further information can be found in table S1. *MYCN* status was assessed as “amplified” for 27 NBL cell lines, “not amplified” for 8 cell lines, and “inconsistent” for 2 cell lines:

1) *MYCN* high copy number [log_2_(CN/2 + 1) ≥ 3] was indicative of “*MYCN*-amplified” status for 23 NBL cell lines and of inconsistent status for 1 cell line (NB17) reported not amplified in Maris and Thiele studies.

2) *MYCN* medium copy number [log_2_(CN/2 + 1) between 1.10 and 3] and high *MYCN* expression [log_2_(TPM + 1) ≥ 5] and/or reported *MYCN*-amplified status was indicative of *MYCN*-amplified for an additional four NBL cell lines.

3) *MYCN* medium copy number and low expression [log_2_(TPM + 1) < 3] and/or reported “not amplified” in publications was indicative of “*MYCN* not-amplified” for four NBL cell lines.

4) *MYCN* low copy number [log_2_(CN/2 + 1) < 1.1], high expression, and reported *MYCN* not-amplified in any study were assessed as inconsistent for one NBL cell line (SKNFI).

The *MYCN* status annotations for NBL cell lines were expanded with the 23Q2 DepMap *MYCN* and *MYC* Chronos dependency scores, *MYC* copy number, gene expression, and with the ssGSEA *z* score for the Helmingler SAGA complex for each NBL cell line.

The genetic differential dependencies enriched in the NBL cell lines versus all other non-NBL cell lines were identified separately for the cell lines with *MYCN*-amplified status and for the cell lines with *MYCN* WT status based on the empirical Bayes linear model implemented in the limma v3.42.2 R package and in the two-class comparison method available from the DepMap Data Explorer interactive platform www.depmap.org, with a cutoff of ≤0.10 for the *P* value corrected for multiple hypothesis testing using the false discovery rate (FDR), (*60*).

### ChIP-seq data analysis

ChIP-seq data analysis was performed in alignment with the ENCODE Consortium standards (www.encodeproject.org/chip-seq/). Quality control tests for unmapped sequences were performed by using the FastQC v.0.11.9 software (www.bioinformatics.babraham.ac.uk/projects/fastqc/). The ChIP sequences were aligned to the GRCh38/hg38 genome and to the spiked-in dm6 *Drosophila melanogaster* genome using bowtie2-2.3.5 (*61*) with the standard options. PCR duplicates were removed with the Picard Mark Duplicates method implemented in the sambamba v0.7.1 tool (*62*). The alignments with a mapping quality score (MAPQ) of <5 were skipped. The mapped reads were normalized in units of reads per kilobase per million [RPKM or rpm/base pair (bp)], and coverage tracks for the RPKM signal were created as bigwig files for bins of size 20 base pairs using the bamCoverage tool available in deepTools v3.5.1 (*63*). The Active Motif spike-in normalization protocol was then applied to each hg38 sample. The dm6 genome-wide counts distribution on 200-bp size bins was computed with the multiBamSummary function available from deepTools v3.5.1. The dm6 uniquely mapped reads were summed up from all the bins with at least 10 mapped dm6 reads. Each hg38 sample was normalized by multiplying the human tag counts with the scaling factor derived from the *Drosophila* reads as ratio between the average of the uniquely mapped dm6 counts across all samples and the dm6 uniquely mapped read counts for that sample. Peak calling was performed against the input control using the model-based MACS2 v2.1.1.20160309 software (*64*), with the significance cutoff FDR of ≤0.01. Area under the curve (AUC) RPKM-normalized signal across genomic regions was computed with the bwtool software (*65*). Each set of MACS2 peaks was curated by removing the binding regions with low area under curve coverage [log_2_(AUC + 1) < 13] and the regions overlapping with the ENCODE black-listed regions for hg38 (available at www.encodeproject.org/annotations/ENCSR636HFF/). Quality control tests for the peaks were performed by using the ChIPQC library available from Bioconductor v3.9 (*66*).

The peaks were annotated with the closest hg38 genes using the annotatePeaks function implemented in the Homer v4.11 platform (*67*) and the GREAT annotation platform (*68*). Binding peaks and normalized binding signal were visualized on the Integrative Genomic Viewer (IGV) v2.12.3 platform (*69*).

Gene promoter regions were defined as the ±3.0-kb intervals around the hg38 gene transcription start site (TSS). Enhancer regions were defined as the H3K27ac binding regions outside TSS ± 3.0-kb gene promoter regions. The SAMtools v1.9 (*70*) and the BEDTools v2.29 suite (*71*) were used to perform various mapping and genomic region analyses (indexing, sorting, intersection, and merging).

For each antibody, the binding sites identified by MACS2 for treatment and control conditions across time points were merged into the antibody’s set of aggregated peaks. A peak by sample counts matrix was created by counting the reads overlapping each aggregated peak with the multiBamSummary tool available from deepTools. The counts matrix was used to perform differential peak analysis. For a specific genomic region, the changes in signal between two conditions were classified as increase, decrease or not significant on the basis of the absolute cutoff of 1.5 for the delta area under curve scores; the significance was quantified on the basis of the cutoff *P* ≤ 0.10 for the differences in the mapped reads by using edgeR v3.36.0 with the glmLRT model (*72*).

Heatmaps of AUC ChIP-seq normalized signal occupancy on genomic regions were created using the computeMatrix and the plotHeatmap tools available in deepTools v3.5.1. The plotProfile tool from deepTools v3.5.1 was used to create metaplots based on the average normalized scores across genomic regions. Differences between metaplots were estimated on the basis of the *t* test with Welch correction (cutoff *P* ≤ 0.05) for the AUC RPKM-normalized signal across the regions for the conditions. Motif enrichment analysis was performed for genomic regions and gene promoters using the Homer v4.11 platform (*67*) and the MEME Suite v5.4.1 platform (find individual motif occurrences method) (*73*) with the adjusted *P* value cutoff of ≤0.01.

### ATAC-seq data analysis

ATAC-seq data analysis was performed in alignment with the ENCODE Consortium standards (www.encodeproject.org/atac-seq/). Briefly, quality control for the paired-end unmapped reads was performed with the FastQC v.0.11.9 software (www.bioinformatics.babraham.ac.uk/projects/fastqc/). The reads were trimmed and filtered for Nextera adapters using Trimmomatic v0.39 (*74*). The trimmed reads were mapped to the hg38 reference genome using bowtie2-2.3.5 (*61*) with the –local –very sensitive –X 2000 options. Bam files were deduplicated using Picard tools (“Picard Toolkit,” 2019, Broad Institute, GitHub Repository, https://broadinstitute.github.io/picard/). Only reads mapping to chromosomes 1 to 22 and chrX with an MAPQ of ≥5 were retained. Reads were shifted with 4 bp on the positive strand and −5 bp on the negative strand using AlignmentSieve available in deepTools v3.5.1 (*63*).

Replicate correlations were computed with the multiBamSummary and bamCorrelate tools available in deepTools v3.5.1 and visualized as dendrograms and in principal components analysis plots. Fragment size distributions were computed with the bamPEFragmentSize tool available in deepTools v3.5.1 and then inspected for quality control. Properly aligned reads for replicates were merged for each condition. Peak calling was performed for properly aligned and for the merged reads with MACS2 v2.1.1.20160309 software (*64*), with the –nomodel, --extsize to fragment length and –q 0.01 options. AUC binding signal for peaks was computed with the bwtool software (*65*). The peaks were annotated with the closest hg38 genes using the annotatePeaks function implemented in the Homer v4.11 platform (*67*) and the GREAT annotation platform (*68*). Gene promoter regions were defined as the ±3.0-kb intervals around the hg38 gene TSS. Enhancer regions were defined as the H3K27ac binding regions outside TSS ± 3.0-kb gene promoter regions.

For each time point, the binding sites identified by MACS2 on merged reads were aggregated into consensus peaks by requiring the AUC binding signal of ≥1000 for each replicate. The consensus peaks for both TADA2B-repressed and control conditions were merged into a set of high-confidence aggregated peaks, separately per each time point. A peak by sample counts matrix was created by counting the reads overlapping each aggregated peak with the multiBamSummary tool available from deepTools v3.5.1. Genome track files were created with the bamCoverage tool available in deepTools v3.5.1 and visualized as heatmaps created with the computeMatrix tool available in deepTools v3.5.1 and with the IGV v2.12.3 platform (*69*).

### RNA-seq data analysis

RNA-seq data analysis was performed in alignment with the ENCODE Consortium standards (www.encodeproject.org/chip-seq/). Quality control tests for unmapped reads were performed based on the FastQC v.0.11.9 software (Babraham Bioinformatics, www.bioinformatics.babraham.ac.uk/projects/fastqc/) and summarized with multiQC v1.9 (*75*). All samples except the *TADA2B* CRISPR KO and sg *LacZ* at 12 days and GSK-699-1 treated at 6, 24, and 72 hours were spiked-in with the ERCC RNA Spike-In Mix1 (Thermo Fisher Scientific, catalog no. 4456740; https://tools.thermofisher.com/content/sfs/manuals/ERCC92.zip). The spiked human reads were mapped to hg38/gencode v30 and to the ERCC genome using STAR v2.7.2b (*76*) with standard parameters --outSAMtype BAM SortedByCoordinate --outSAMunmapped None --outSAMattributes NH HI NM MD AS XS --outReadsUnmapped Fastx --outSAMstrandField intronMotif --quantMode TranscriptomeSAM GeneCounts --quantTranscriptomeBan IndelSoftclipSingleend. Quality control for the mapped reads and that for replicate reproducibility were performed using SARTools v1.7.3 (*77*). Gene level reads were summarized by counting the reads that overlapped the hg38/gencode v30 gene exons and separated the ERCC-annotated genes using the featureCounts v1.6.3 method implemented in the Subread v2.0.0 package (http://subread.sourceforge.net) (*78*). The erccdashboard R package (*79*) available from Bioconductor v3.9 was used to estimate the ERCC spiked-in variability in transcriptional performance. Gene counts were normalized and used to quantify the differential genes between the experimental and control conditions using DESeq2 v1.32.0 (*80*). Gene expression was estimated on the basis of the log_2_(TPM + 1) scores for normalized reads (*81*). The expressed genes were identified as the genes with the maximum log_2_(TPM + 1) expression of ≥1 across all conditions. The gene differentiability was assessed with DESeq2 based on the robust shrunken log_2_ fold change scores and the approximate posterior estimation for generalized linear model coefficients (apeglm v1.6) method for effect size (*80*). The cutoffs for gene differentiability were |fold change expression| ≥ 1.5 and adjusted *P* ≤ 0.10. Heatmaps for the visualization of transcriptional changes were created by using the Morpheus software platform (https://software.broadinstitute.org/morpheus/) based on the log_2_(fold change) expression data.

### Gene set enrichment analyses

GSEA v4.2.0 software (*82*, *83*) was used to identify functional associations of the molecular phenotypes induced by TADA2B loss with the MSigDB v7.4 collections of gene sets (*84*), including 50 hallmark gene sets (h), 6290 curated pathways and experimental gene sets (c2), and 7481 Gene Ontology/Biological Processes gene sets (c5), with CORUM v4.1 (*85*) and with the van Groningen *et al.* (*86*) neuroblastoma lineage differentiation signatures and several published MYCN and MYC target gene set signatures as described in table S3. For each experimental comparison, the hg38/gencode v30–expressed genes were ranked on the basis of the expression fold change in treated versus control phenotypes. The goal of GSEA was to identify the gene sets that are distributed at the top or at the bottom of the ranked list of genes based on the Kolmogorov-Smirnov enrichment test. Gene sets with an absolute normalized enrichment score (NES) of ≥1.3, a nominal *P* ≤ 0.05 and an FDR of ≤0.25 for the Kolmogorov-Smirnov test were considered significant hits. The results were visualized on volcano plots for the NES versus −log_10_(*P*) and on GSEA plots. In addition, the functional associations of the molecular phenotypes were explored with the ssGSEA method (*87*) based on the Bioconductor GSVA v1.40.1 implementation (*88*). ssGSEA is an extension of GSEA that calculates separate enrichment scores for each pairing of a sample and gene set. Each ssGSEA enrichment score represents the degree to which the genes in a particular gene set have coordinately increased or decreased expression within a sample. Overlapping enrichment analysis for the genes with significantly altered expression induced by TADA2B loss was performed against the MSigDB v7.4 gene set collections. Significant enrichments were quantified on the basis of the hypergeometric test (*P* ≤ 0.05 and FDR ≤ 0.05) and size overlap of ≥5.

## Supplementary Materials

### This PDF file includes:

Figs. S1 to S9 Legends for tables S1 to S4 Uncropped Western blots
