# Supplementary material for: The KAT module of the SAGA complex maintains the oncogenic gene expression program in MYCN-amplified neuroblastoma
Source: Sci Adv. 2024 May 31;10(22):eadm9449. doi: 10.1126/sciadv.adm9449 (PMC11141635; doi:10.1126/sciadv.adm9449)
Supplement: Supplementary file 1 — Figs. S1 to S9 Legends for tables S1 to S4 Uncropped Western blots [file sciadv.adm9449_sm.pdf]

Science Advances
AAAS

Supplementary Materials for

The KAT module of the SAGA complex maintains the oncogenic gene expression program in *MYCN*-amplified neuroblastoma

Clare F. Malone *et al.*

Corresponding author: Kimberly Stegmaier, kimberly_stegmaier@dfci.harvard.edu

Sci. Adv. **10**, eadm9449 (2024)
DOI: 10.1126/sciadv.adm9449

The PDF file includes:

Figs. S1 to S9
Legends for tables S1 to S4
Uncropped Western blots

Other Supplementary Material for this manuscript includes the following:

Tables S1 to S4

Supplemental Figure 1

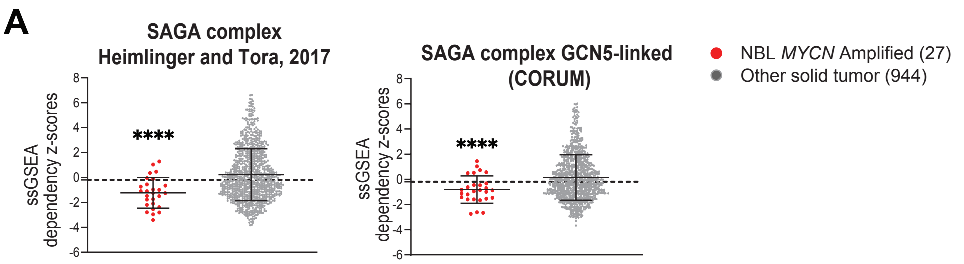

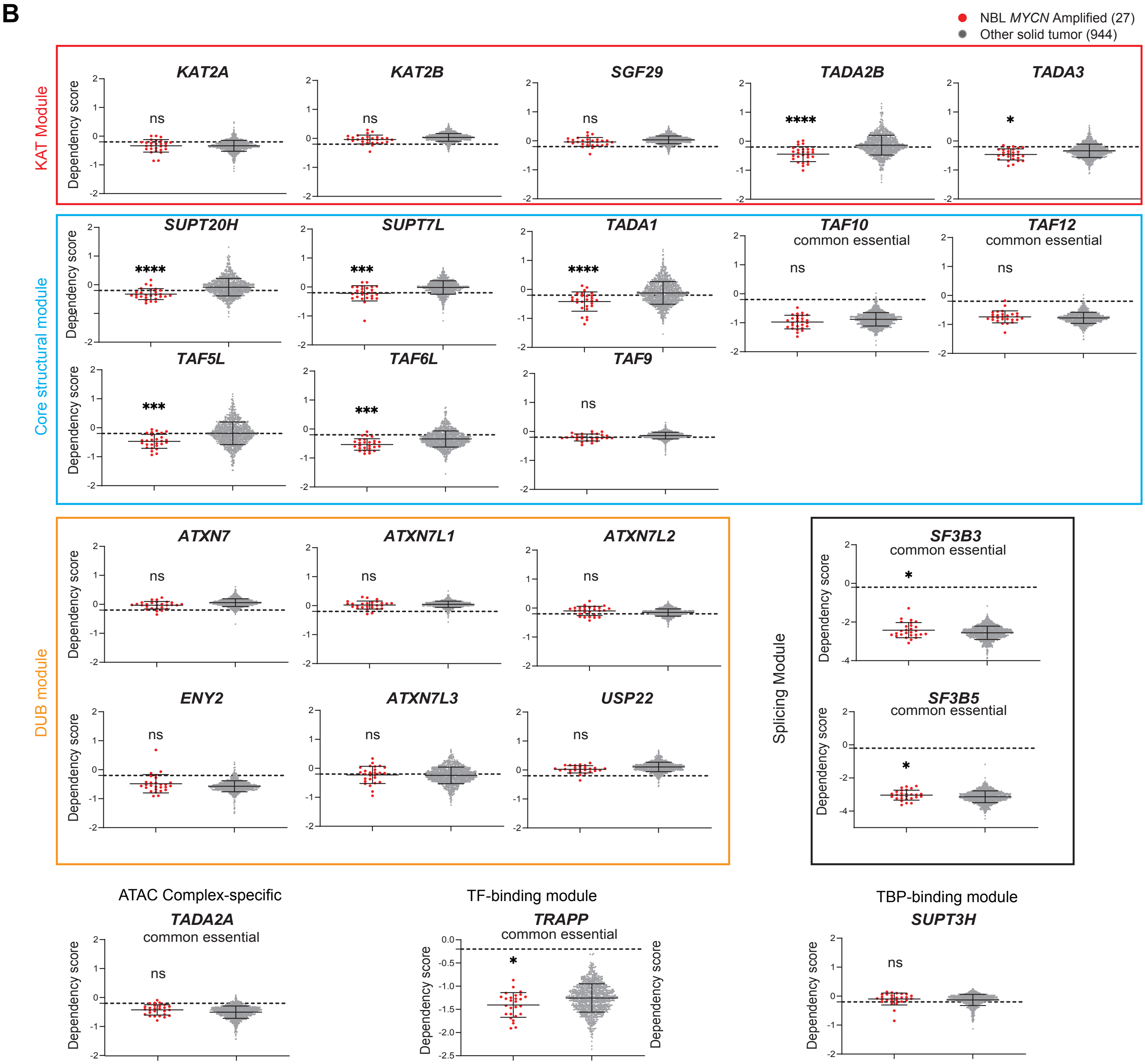

Supplemental Figure S1. SAGA complex members are selective dependencies in *MYCN*-amplified neuroblastoma.

A) Mean + SD scatterplots presenting the ssGSEA dependency z-scores for the SAGA complex annotated by “SAGA COMPLEX GCN5-LINKED” in CORUM and the SAGA complex defined in Helmlinger and Tora, 2017 *(18)* across *MYCN*-amplified neuroblastoma and non-neuroblastoma solid tumor cell lines available in the 23Q2 CRISPR (Public+Score, Chronos) data. The number of cell lines per group is shown in parentheses. Differential dependency vs. non-neuroblastoma cell lines, unpaired t-test with Welch’s correction *** $p < 0.001$. **B)** Mean + SD scatterplots presenting Chronos dependency scores for SAGA complex genes and for the ATAC gene *TADA2A* across *MYCN*-amplified neuroblastoma and non-neuroblastoma solid tumor cell lines available in the 23Q2 CRISPR (Public+Score, Chronos) data. The number of cell lines per group is shown in parentheses. Differential dependency vs. non-neuroblastoma cell lines, unpaired t-test with Welch’s correction *** $p < 0.001$, * $p < 0.05$, ns= not significant.

Supplemental Figure 2

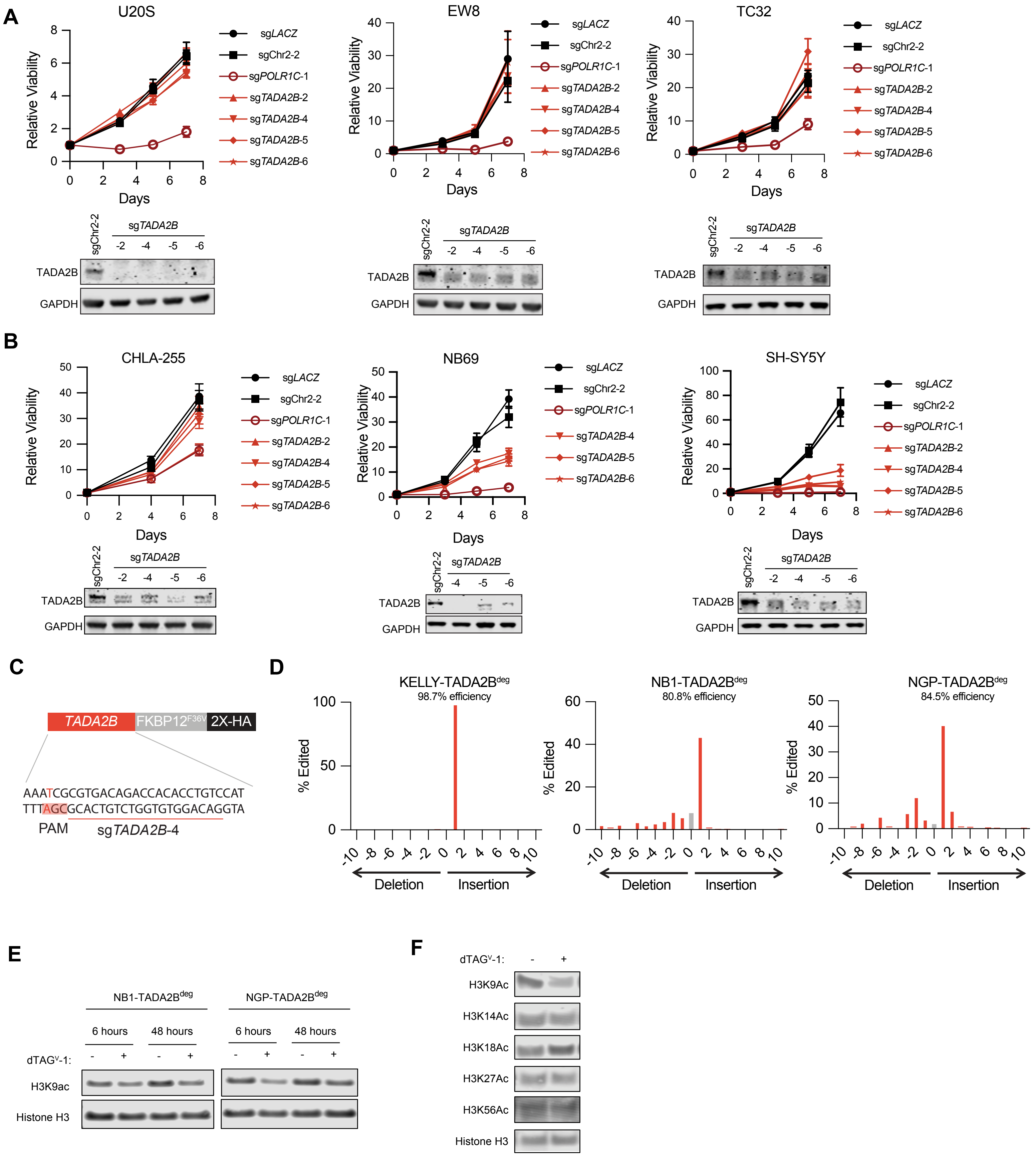

Supplemental Figure S2. TADA2B is a dependency in *MYCN*-amplified neuroblastoma.

A) U20S (left, osteosarcoma), EW8 (middle, Ewing sarcoma), and TC32 (right, Ewing sarcoma) cells were infected with sgRNAs targeting *LACZ* or a gene desert on chromosome 2 (sgChr2-2) as negative controls (black), *TADA2B* (red), or the common essential gene *POLR1C* (dark red). Five days after infection, cell lines were plated and assessed for viability on days 3, 5, and 7 as compared to day 0 of plating. Below, western blots showing TADA2B expression in sgChr2-2 control and *TADA2B* knockout. GAPDH is included as a loading control. **B)** CHLA-255 (left), NB69 (middle), and SH-SY5Y (right) non MYCN-amplified neuroblastoma cells were infected with sgRNAs targeting *LACZ* or a gene desert on chromosome 2 (sgChr2-2) as negative controls (black), *TADA2B* (red), or the common essential gene *POLR1C* (dark red). Five days after infection, cell lines were plated and assessed for viability on days 3, 5, and 7 as compared to day 0 of plating. Below, western blots showing TADA2B expression in sgChr2-2 control and *TADA2B* knockout. GAPDH is included as a loading control. **C)** Diagram of exogenous *TADA2B* with FKBP12^{F36V} and HA tags. Below, the site of the sgRNA used to knock out endogenous *TADA2B* is shown, with the silent point mutation in the exogenous *TADA2B* PAM site highlighted in red, rendering it resistant to CRISPR editing. **D)** Endogenous *TADA2B* editing in the KELLY-TADA2B^{deg}, NB1-TADA2B^{deg}, and NGP-TADA2B^{deg} lines was assessed using TIDE sequencing. The bar graphs show the percentage of sequences with insertions or deletions at the given location relative to the cut site, and at top the overall editing efficiency is shown. **E)** NB1-TADA2B^{deg} and NGP-TADA2B^{deg} cells were treated with DMSO or 500 nM dTAG^{V}-1 for 6 or 48 hours as indicated, and then histones were extracted. H3K9 acetylation levels are shown and Histone H3 serves as a loading control. **F)** KELLY-TADA2B^{deg} cells were treated with DMSO or 500 nM dTAG^{V}-1 for 48 hours as indicated, and then histones were extracted. H3K9, H3K14, H3K18, H3K27, H3K56 acetylation levels are shown. Histone H3 serves as a loading control.

Supplemental Figure S3

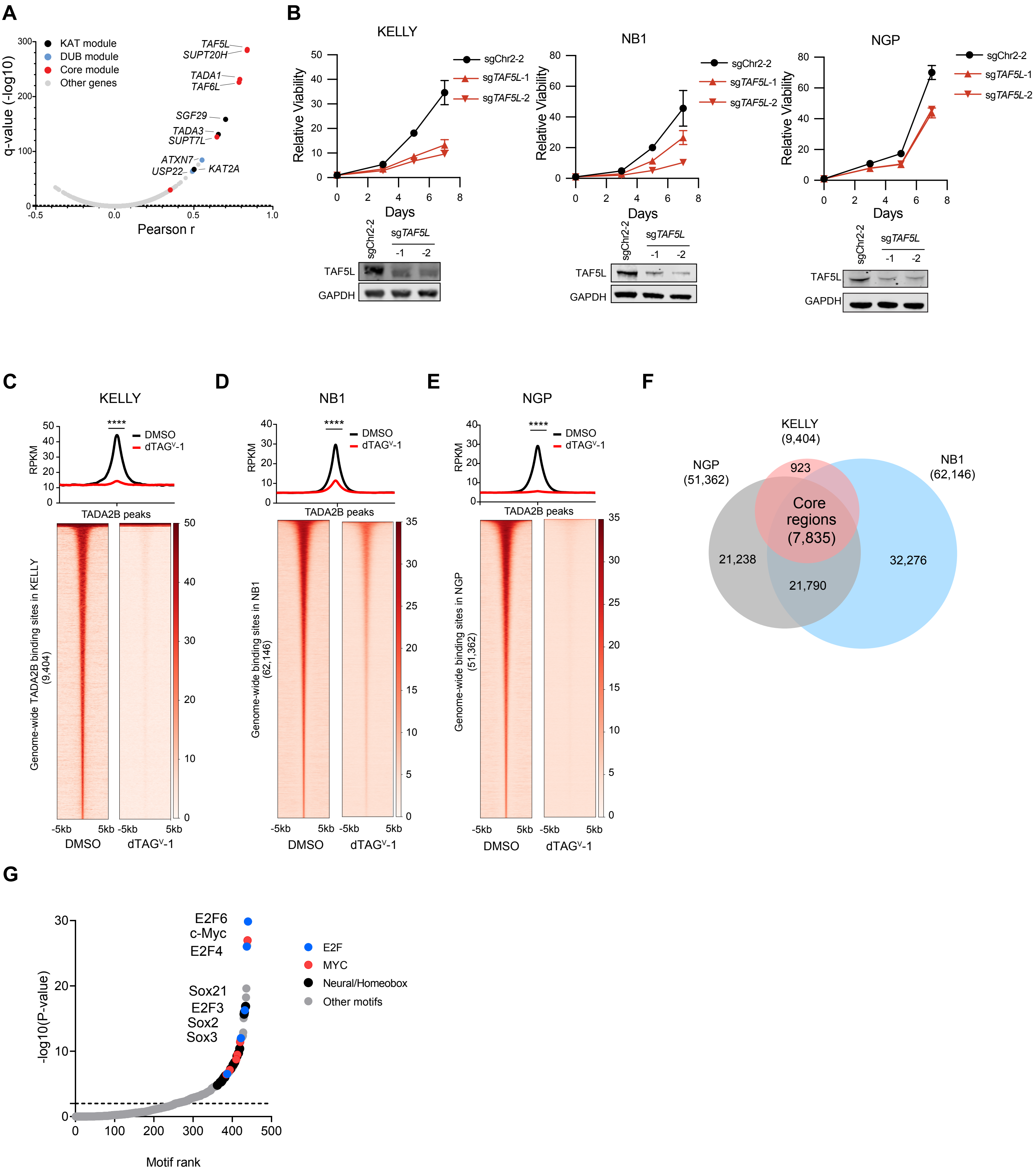

Supplemental Figure S3 TADA2B co-localizes with MYCN, H3K9Ac, H3K27Ac, and open chromatin at promoters of actively transcribed genes

A) Volcano plot showing the Pearson r correlation scores of *TADA2B* genetic dependency against all other genes for all cell lines in 23Q2 DepMap data. Relevant SAGA modules including the KAT (black), DUB (blue), and red (core) are indicated and specific genes labelled. **B)** KELLY (left), NB1 (middle), or NGP (right) cells were infected with sgRNAs targeting a gene desert on chromosome 2 (sgChr2-2) as negative control (black) or *TAF5L* (red). Five days after infection, cell lines were plated and assessed for viability on days 3, 5, and 7 as compared to day 0 of plating. Below, western blots showing TAF5L expression in sgChr2-2 control and *TAF5L* knockout. GAPDH is included as a loading control. **C)** Genome-wide heatmaps depicting the effect of TADA2B degradation on anti-TADA2B-HA ChIP-Seq six hours after treatment with DMSO or 500 nM $dTAG^{V}$-1 on KELLY-$TADA2B^{deg}$. Heatmaps are centered on TADA2B peaks. Regions are ranked based on DMSO signal. At top, read density metaplots showing average RPKM normalized signal for TADA2B in DMSO (black) and $dTAG^{V}$-1 (red) treated KELLY-$TADA2B^{deg}$ cells. Differential read density in $dTAG^{V}$-1 versus DMSO conditions estimated based on unpaired t-test with Welch's correction, ****p < 0.0001. **D)** As in **C**, in NB1 -$TADA2B^{deg}$ cells. **E)** As in **C,** in NGP-$TADA2B^{deg}$ cells. **F)** Venn diagram depicting the overlap between the TADA2B binding regions identified in KELLY-$TADA2B^{deg}$, NB1 -$TADA2B^{deg}$ and NGP-$TADA2B^{deg}$ cell lines. The significance of the three region sets overlap estimated based on the super exact test one-tailed cumulative probability function; **** p < 0.0001. **G)** Hockey plot highlighting representative motifs enriched at the promoters of the nearest gene targets for core TADA2B binding regions (homer v4.11, FDR ≤ e-05).

Supplemental Figure S4

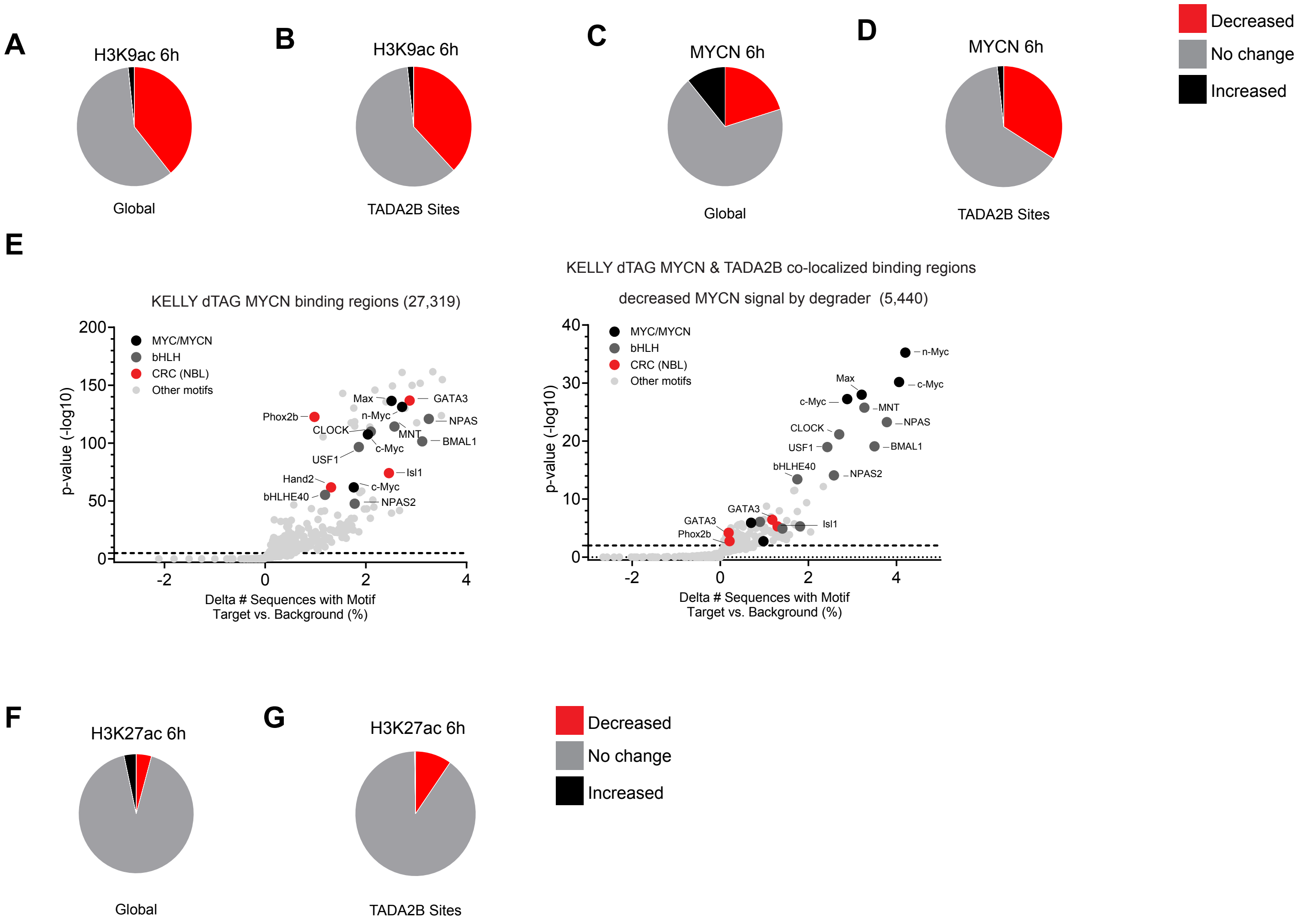

Supplemental Figure S4 Loss of TADA2B results in rapid and global loss of H3K9ac, MYCN, and H3K27ac

A) Pie chart depicting the distribution of all H3K9ac binding sites with decreased (red), not significantly changed (grey) and increased H3K9ac signal (black) in dTAG^{V}-1 vs. DMSO conditions at 6 hours **B)** Pie chart depicting the distribution of H3K9ac changes at TADA2B binding sites with decreased (red), not significantly changed (grey) and increased H3K9ac signal (black) in dTAG^{V}-1 vs. DMSO conditions at 6 hours. **C)** Pie chart depicting the distribution of all MYCN binding sites with decreased (red), not significantly changed (grey) and increased MYCN signal (black) in dTAG^{V}-1 vs. DMSO conditions at 6 hours. **D)** Pie chart depicting the distribution of MYCN changes at TADA2B binding sites with decreased (red), not significantly changed (grey) and increased MYCN binding (black) in dTAG^{V}-1 vs. DMSO conditions at 6 hours. **E)** Volcano plots showing the top enriched motifs for global MYCN binding (left) or TADA2B and MYCN co-bound peaks that demonstrated reduced MYCN binding with TADA2B degradation (right) (homer v4.11, FDR ≤ e-05). Motif categories including core regulatory circuitry (CRC), basic helix loop helix (bHLH), and MYC/MYCN are included. **F)** Pie chart depicting the distribution of all H3K27ac sites with decreased (red), not significantly changed (grey) and increased H3K27ac signal (black) in dTAG^{V}-1 vs. DMSO conditions at 6 hours. **G)** Pie chart depicting the distribution of the H3K27ac changes at TADA2B binding sites with decreased (red), not significantly changed (grey) and increased H3K27ac signal (black) in dTAG^{V}-1 vs. DMSO conditions at 6 hours.

Supplemental Figure S5

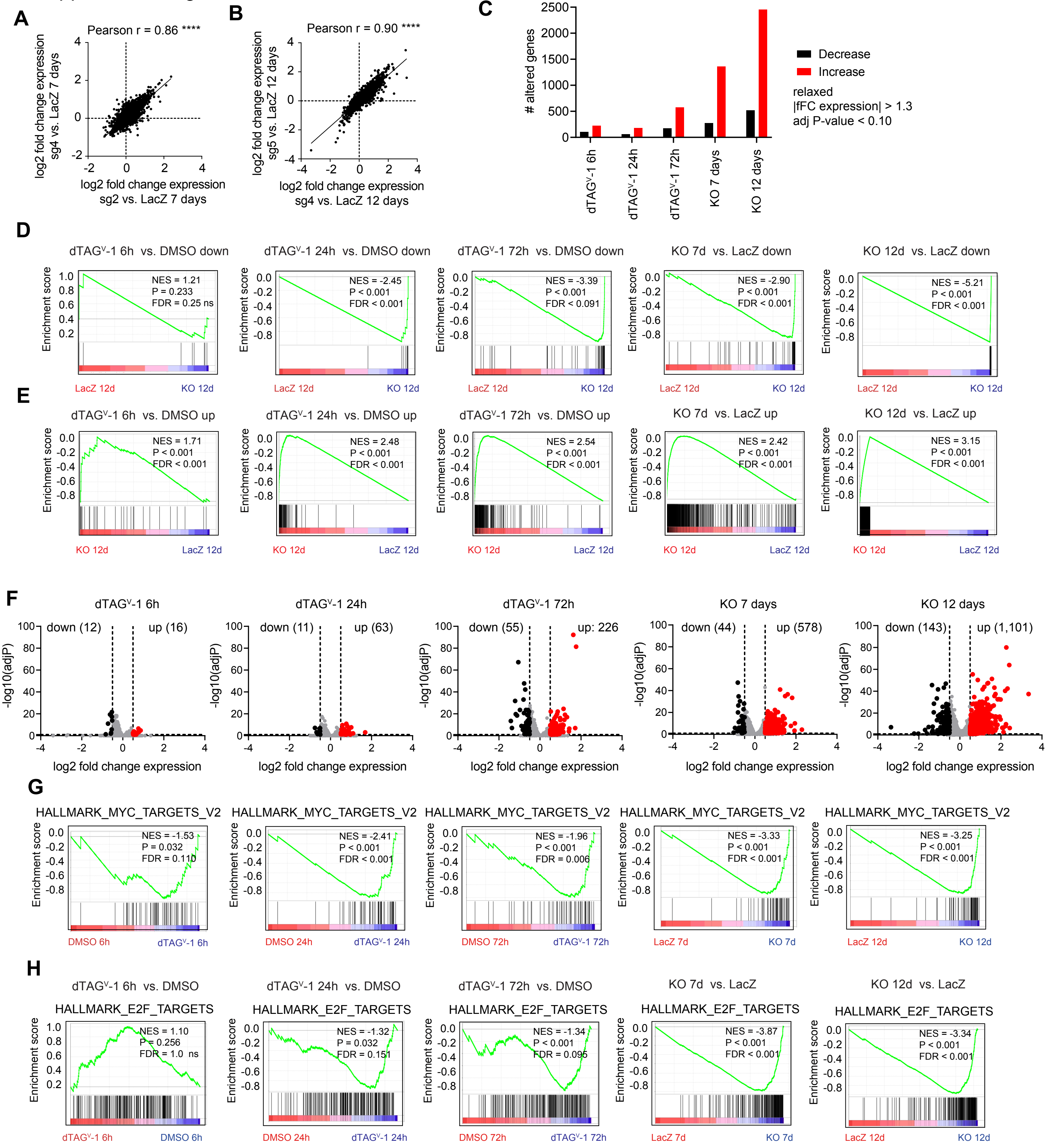

Supplemental Figure S5. TADA2B loss leads to modest gene expression changes

A) Scatter plots demonstrating the correlation of genome-wide gene expression changes induced by the paired guides used for CRISPR KO at 7 days **B)** As in **A,** for CRISPR KO at 12 days. **C)** Bar plots depicting the number of differentially expressed genes induced by TADA2B loss, estimated based on relaxed cut-offs (DESeq2 adj $p \leq 0.10$, |fold change expression| ≥ 1.3). **D-E)** GSEA plots demonstrating the enrichment of the TADA2B loss gene signatures for $dTAG^{V}$-1 and CRISPR KO conditions (decrease **D**, increase **E)** in the genome-wide expression changes induced by sgTADA2B vs. sgLACZ at 12 days. Normalized enrichment score, p value, and false discovery rate (FDR) are indicated in each plot. **F)** Volcano plots depicting the differentially expressed genes induced by TADA2B loss in degradation and CRISPR KO conditions. Highlighted are genes with decreased expression (black) and genes with increased expression (red). Significance DESeq2 adj $p \leq 0.10$, |fold change expression| ≥ 1.5. **G)** GSEA plots demonstrating the enrichment of the genome-wide TADA2B loss signatures in $dTAG^{V}$-1 and CRISPR KO conditions vs. Hallmark_MYC_Targets_V2. Normalized enrichment score, p value, and false discovery rate (FDR) are indicated in each plot. **H)** As in **G,** for Hallmark_E2F_targets.

Supplemental Figure S6

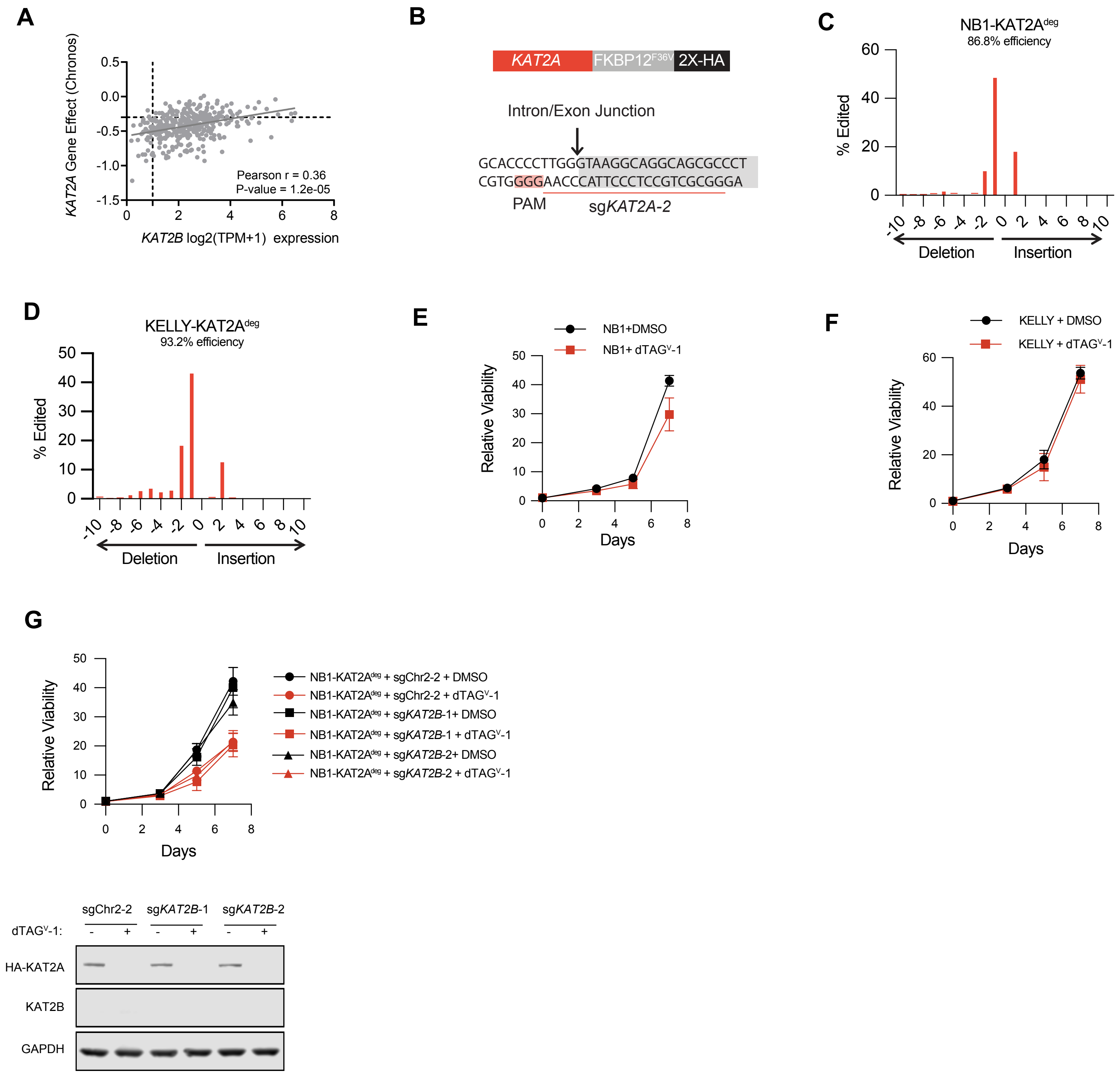

Supplemental Figure S6. KAT2A and KAT2B are functionally redundant in neuroblastoma. **A)** Scatter dot plot depicting the linear association between the *KAT2A* dependency gene effect and the *KAT2B* log2(TPM+1) gene expression across all *TADA2B* dependent cell lines in the 23Q2 DepMap data. *TADA2B* dependency per cell line estimated based on the gene effect Chronos dependency score ≤ -0.30 in the CRISPR (Public+Score, Chronos) data. Significance: F-test for rejecting the null hypothesis that linear regression slope is 0, p-value cut-off ≤ 0.01. **B)** Diagram of exogenous *KAT2A* with C-terminal FKBP12^{F36V} and HA tags. Below, the sequence of the endogenous *KAT2A* targeted by sgKAT2A-2 is shown, with the exon shown in grey and the intron, where the PAM site is, in white. **C)** TIDE sequencing was performed to confirm endogenous editing in the NB1 and KELLY KAT2A^{deg} lines. The bar plots show the percent of sequences with insertions or deletions at the given position relative to the PAM site. At top, the overall editing efficiency is shown. **D)** As in **C,** for KELLY-KAT2A^{deg} cells. **E)** Parental NB1s were treated with DMSO or 1 µM dTAG^{V}-1 and viability, relative to day 0, was assessed using CellTiter-Glo. Days are shown on the x-axis, and relative viability is on the y-axis. Mean and SD are shown. **F)** Kelly cells were treated as in **E**. Mean and SD are shown. **G)** NB1 cells with endogenous *KAT2A* knocked out and expressing degron-tagged exogenous *KAT2A* were infected with sgRNAs targeting a gene desert on chromosome 2 (sgChr2-2) or *KAT2B*, as indicated. Cells were then treated with DMSO (black) or dTAG^{V}-1 (red) and relative viability was assessed using CellTiter-Glo. Mean and SD are shown. Below, western blot of exogenous KAT2A (HA) levels after dTAG^{V}-1 treatment**.** Western blot showing KAT2A-HA and KAT2B expression with GAPDH loading control is shown below for cell lines used in viability analysis.

Supplemental Figure S7

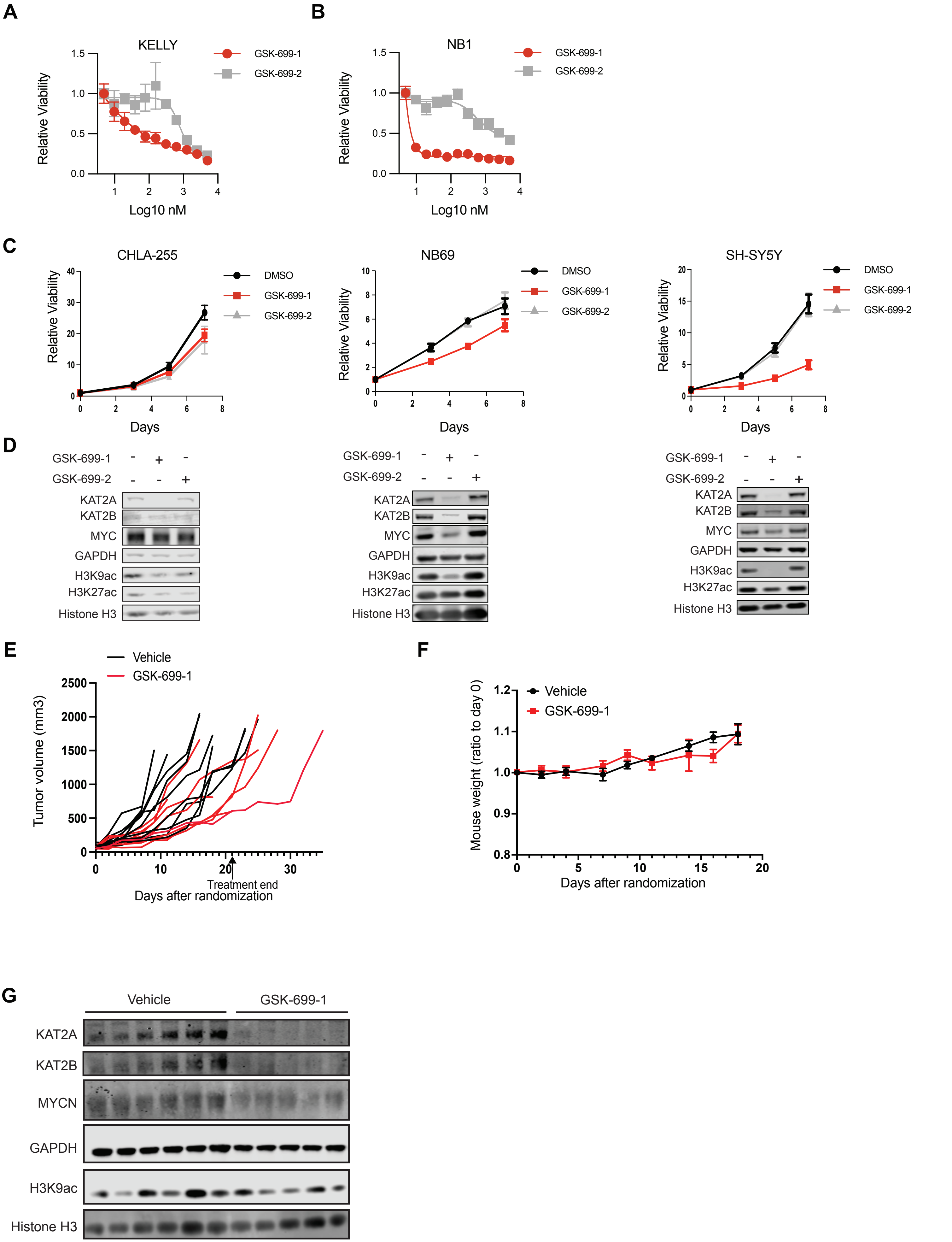

Supplemental Figure S7. KAT2A and KAT2B degradation is efficacious in neuroblastoma. A) Concentration response curves showing cellular viability in Kelly for active GSK-699-1 and inactive GSK-699-1 compounds as determined by CellTiter-Glo on day 5 and normalized to DMSO control. **B)** As in A, for NB1 cells. Data are shown as mean + SD**. C)** Relative viability, assessed by CellTiter-Glo and normalized to day 0, is shown on the y-axis for CHLA-255 (left), NB69 (middle), and SH-SY5Y (right) cells treated with vehicle, 100 nM GSK-699-1, or 100 nM GSK-699-2 for 3, 5, or 7 days. Mean and SD are shown. **D)** Western blots corresponding to the experiments in panel C showing expression of KAT2A, KAT2B, MYCN, H3K9ac, or H3K27ac in CHLA-255 (left), NB69 (middle), or SH-SY5Y (right) cells treated for 24 hours with vehicle, GSK-699-1, or GSK-699-2. GAPDH and Histone H3 are shown as loading controls. **E**) Spaghetti plots showing tumor volume for each individual mouse injected with Kelly xenografts and treated with either vehicle or 50 mg / kg IP daily of GSK-699-1. Vehicle is indicated in black, and GSK-699-1 is indicated in red. Arrow denotes the day that treatment stopped. **F)** Mean + SD Scatterplot showing mouse weights normalized to day 0 for vehicle (n=5) or GSK-699-1 (n=7) cohorts that received at least 18 days of treatment. **G)** Western blot of KAT2A, KAT2B, MYCN, and H3K9ac expression in Kelly xenografts from panel D at study endpoint. GAPDH and Histone H3 serve as loading controls.

Supplemental Figure S8

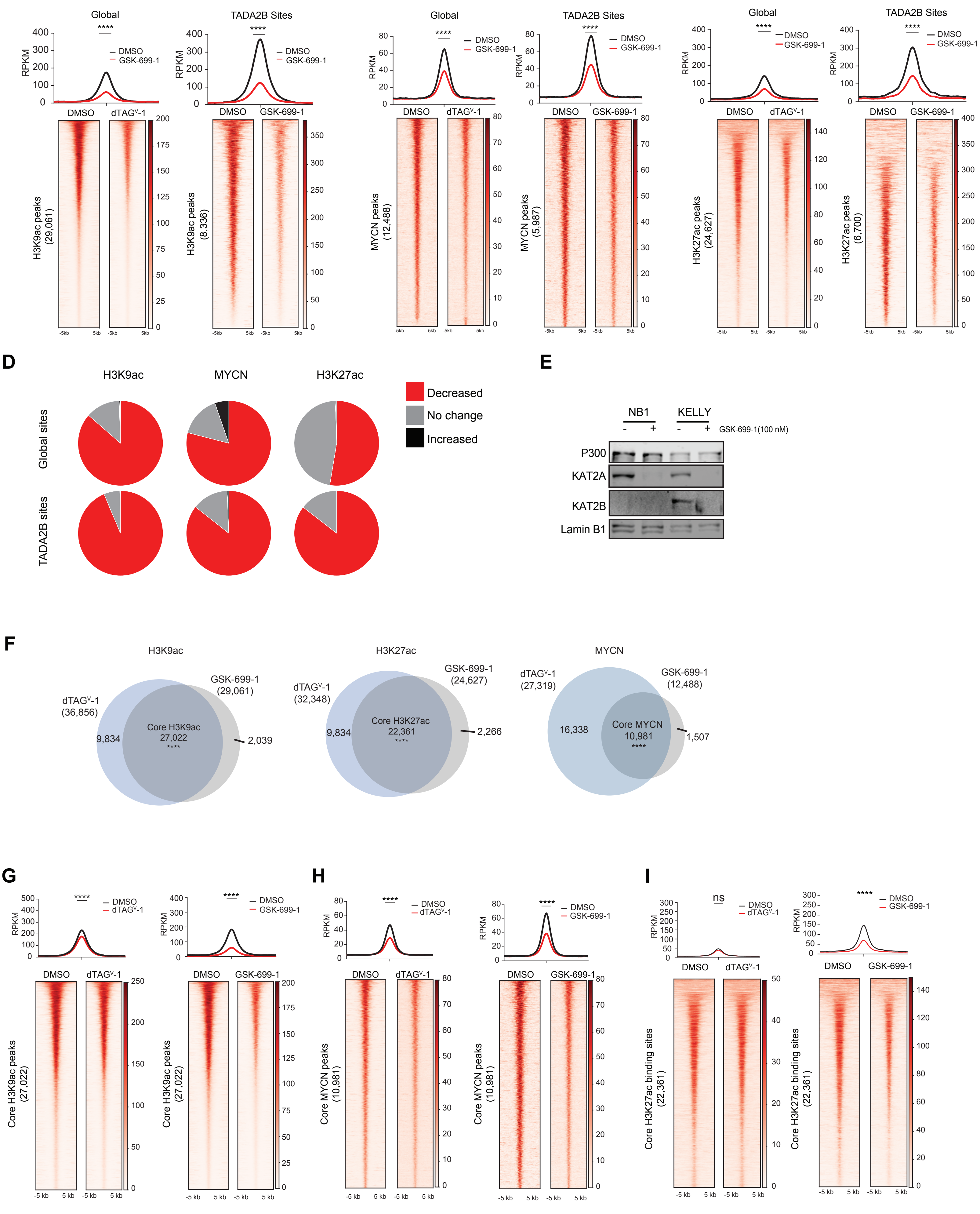

Supplemental Figure S8. Integrated ChIP-sequencing reveals regulation of SAGA-regulated genes with GSK-699-1 treatment

A-C) Genome-wide heatmaps of H3K9ac (**A**), MYCN (**B**), or H3K27ac (**C**) in KELLY cells treated with DMSO or 100 nM GSK-699-1 for 6 hours. Heatmaps are depicted on the universe of merged binding sites across conditions (global; left) or specifically at sites identified as TADA2B-bound (TADA2B sites; right). The regions are ranked based on the binding signal in DMSO. At top, read density metaplots showing average RPKM normalized signal for each of H3K9ac, MYCN, or H3K27ac across the universe of binding sites depicted below in DMSO (black) and 100 nM GSK-699-1 (red) treated KELLY cells. Differential read density between DMSO and GSK-699-1 conditions estimated based on unpaired t-test with Welch's correction, **** $p < 0.0001$. **D)** Pie charts depicting the distribution of all H3K9ac (left), MYCN (middle), or H3K27ac (right) binding sites with decreased (red), not significantly changed (grey) and increased signal (black) in GSK-699-1 treatment vs. DMSO conditions at 6 hours. **E)** Western blot showing expression of P300, KAT2A, KAT2B, and Lamin B1 in nuclear extracts of GSK-699-1 treated NB1 or KELLY cells. **F)** Venn diagrams showing the overlap of peaks detected for each antibody between KELLY-TADA2B^{deg} and GSK-699-1 ChIP-sequencing data sets. Overlapping peaks between data sets were determined as "global core peaks" and used for all subsequent analysis. **G-I)** Genome-wide heatmaps of H3K9ac **(G)**, MYCN **(H)**, or H3K27ac **(I)** in either KELLY-TADA2B^{deg} cells treated with DMSO or 500 nM dTAG^{V}-1 for 6 hours (left) or KELLY cells treated with DMSO or 100 nM GSK-699-1 for 6 hours (right). Heatmaps are depicted on the universe of core sites. The regions are ranked based on the binding signal in DMSO. At top, read density metaplots showing average RPKM normalized signal for each of H3K9ac, MYCN, or H3K27ac across the universe of binding sites depicted below in DMSO (black) and either 500 nM dTAG^{V}-1 or 100 nM GSK-699-1 (red) treated cells. Differential read density between conditions estimated based on unpaired t-test with Welch's correction, **** $p < 0.0001$, ns - not significant.

Supplemental Figure S9

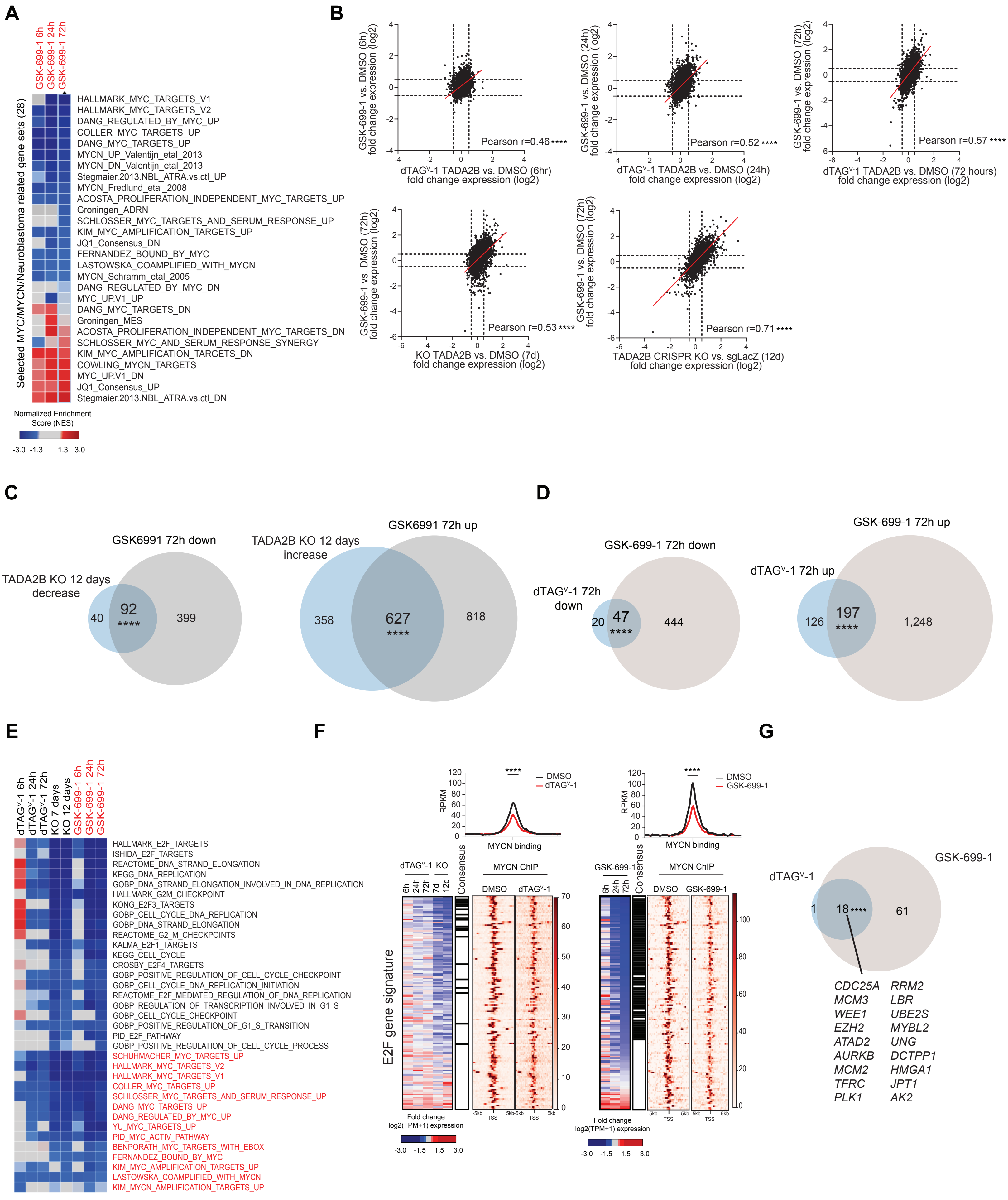

Supplemental Figure S9. Integrated ChIP- and RNA-sequencing reveals regulation of SAGA-controlled genes with GSK-699-1 treatment

A) Heatmap for ssGSEA normalized enrichment scores for MYC and MYCN compendia of gene sets associated with GSK-699-1 vs. DMSO treatment. **B)** Scatterplots showing the correlation of gene expression changes for GSK-699-1 at 6 hours as compared against dTAG^{V}-1 at 6 hours (top left), GSK-699-1 at 24 hours as compared against dTAG^{V}-1 at 24 hours (top middle), GSK-699-1 at 72 hours as compared to dTAG^{V}-1 at 72 hours (top right), GSK-699-1 at 72 hours as compared against *TADA2B* knockout at 7 days (bottom left), and GSK-699-1 at 72 hours as compared against *TADA2B* knockout at 12 days (bottom right). Pearson correlations are shown. Significance was determined by F-test. **C-D)** Venn diagrams showing the overlap of significantly downregulated (left) or upregulated (right) genes between 72 hours treatment with GSK-699-1 and *TADA2B* knockout (12 days) **(C)** or 72 hours treatment with dTAG^{V}-1 **(D)** as determined by RNA-sequencing (DESeq2 adj $p \leq 0.10$, |fold change expression| ≥ 1.5). **E)** Heatmap for ssGSEA normalized enrichment scores for MYC and E2F-related gene sets in the functional categories associated with expression across KELLY-TADA2B^{deg} and GSK-699-1 data sets. Color scale bars are shown at the bottom. Heatmap is sorted based on the GSK-699-1 72-hour time point and organized based on hierarchical clustering. **F)** Integrated heatmap showing RNA-expression across experiments and the corresponding enrichment for MYCN by ChIP-sequencing in vehicle or dTAG^{V}-1 (left) or GSK-699-1 (right) treated cells for the E2F-related gene signatures. Black bars indicate consensus genes for which there was downregulation of the gene by RNA-sequencing and loss of MYCN binding by ChIP-sequencing. Heatmaps are shown with 5kb up- and downstream of the transcriptional start site (TSS). Above, read density metaplots showing average RPKM normalized signal for MYCN across the universe of binding sites depicted below in DMSO (black) and either 500 nM dTAG^{V}-1 or 100 nM GSK-699-1 (red) treated cells. Differential read density between conditions estimated based on unpaired t-test with Welch's correction, ****

$p < 0.0001$, **G)** Venn diagram showing the number of unique and overlapping consensus genes as determined for each treatment in panel F. Overlapping genes are indicated. Significance was determined by Fisher's exact test, **** $p < 0.0001$.

Supplementary Tables

Supplemental Table S1. MYCN curated status for the 37 neuroblastoma cell lines available in the 23Q2 DepMap CRISPR (Public+Score, Chronos) database.

Supplemental Table S2. GSEA results for the enrichments of the MYCN-amplified neuroblastoma differential dependency in the CRISPR (DepMap Public 23Q2+Score, Chronos) database vs the human CORUM v4.1 protein complexes.

Supplemental Table S3. Description of the MYCN related gene sets depicted in Figure 5F.

Supplemental Table S4. MYC/N and E2F metagene integrative data analysis, related to Figures 8K and L. Gene sets contributing to the metagene, TADA2B target genes and consensuses genes are indicated by black dots.

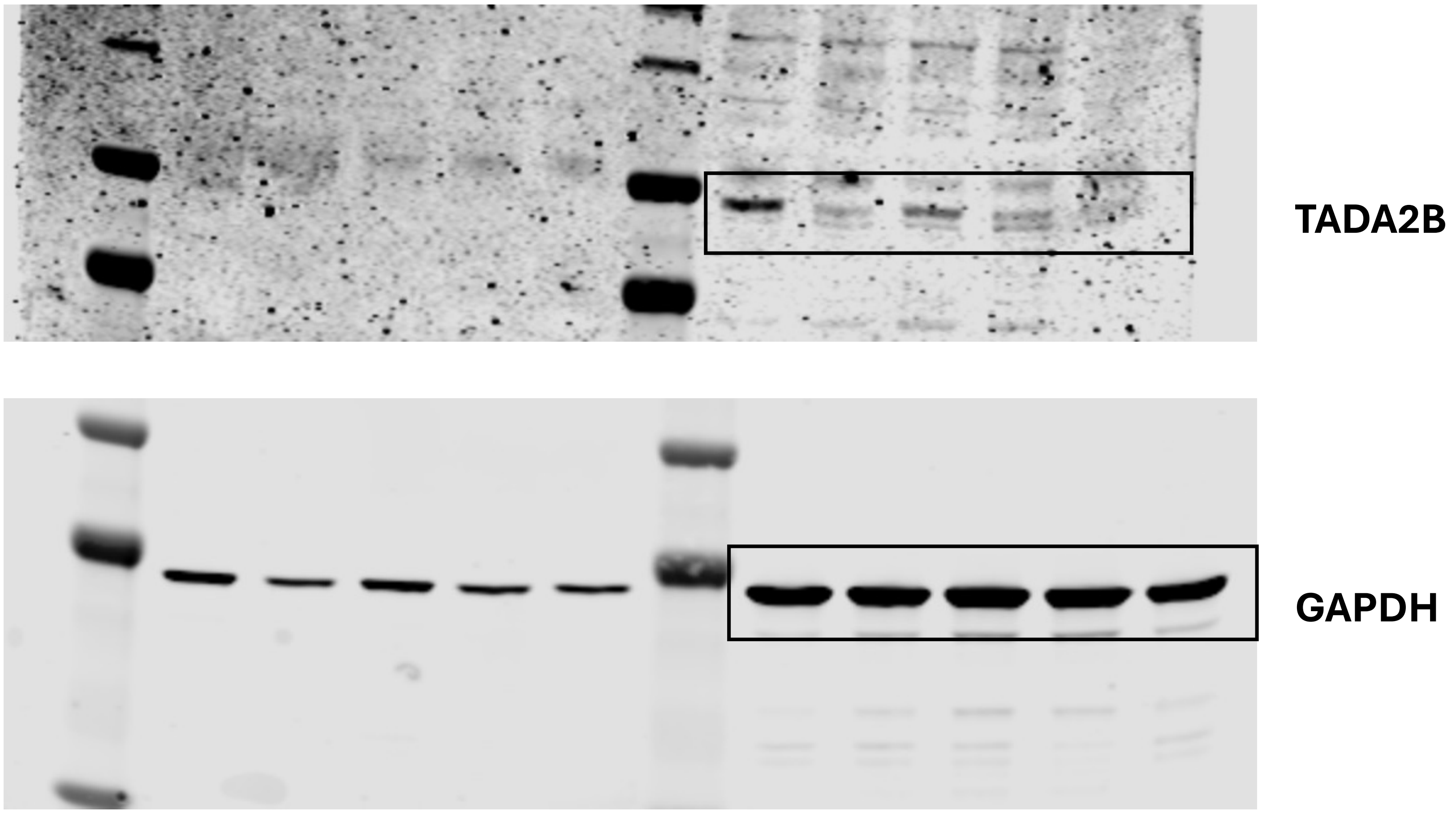

Figure 2A – NB1

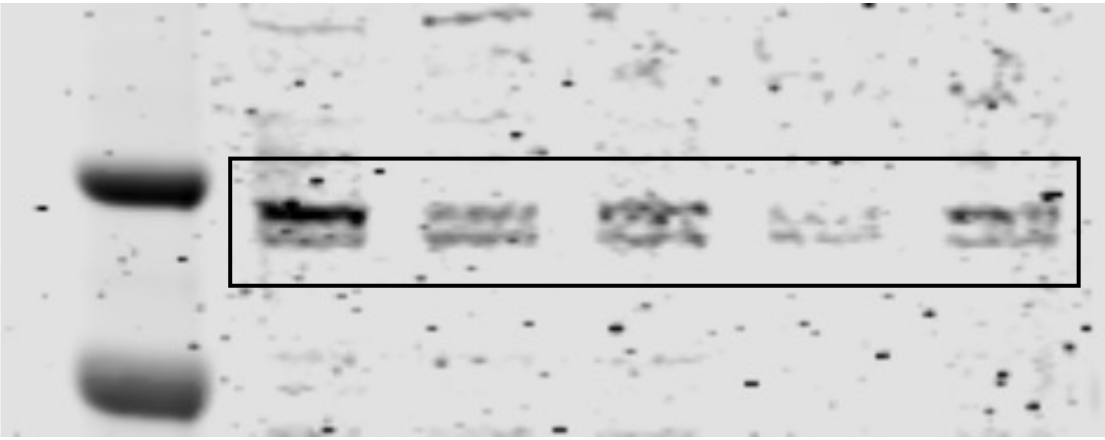

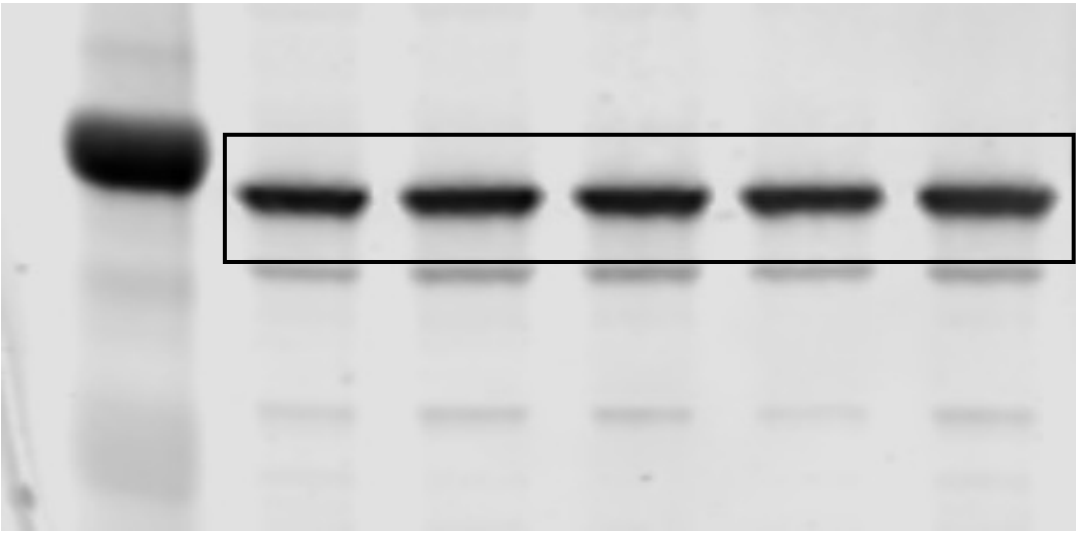

Supplemental Figure 2B CHLA-255
